# Effects of aerobic or resistance exercise on sleep and cancer-related fatigue in patients with breast cancer during or after neoadjuvant chemotherapy: a 3-arm randomized controlled trial

**DOI:** 10.1186/s12916-026-04669-3

**Published:** 2026-01-28

**Authors:** Alexander Haussmann, Martina E. Schmidt, Siri Goldschmidt, Anouk E. Hiensch, Joachim Wiskemann, Karen Steindorf

**Affiliations:** 1 Division of Physical Activity, Cancer Prevention and Survivorship, German Cancer Research Center (DKFZ) and National Center for Tumor Diseases (NCT) Heidelberg, a partnership between DKFZ and University Medical Center Heidelberg, Im Neuenheimer Feld 581, Heidelberg, 69120 Germany; 2 Section of Health Care Research and Rehabilitation Research, Institute of Medical Biometry and Statistics, Faculty of Medicine, Medical Center, University of Freiburg, Hugstetter Str. 49, Freiburg, 79106 Germany; 3 University Medical Center Utrecht, Utrecht University, P.O. Box 85500, Utrecht, GA 3508 The Netherlands; 4 Division of Medical Oncology, University Clinic Heidelberg and National Center for Tumor Diseases (NCT) Heidelberg, a partnership between DKFZ and University Medical Center Heidelberg, Im Neuenheimer Feld 460, Heidelberg, 69120 Germany

**Keywords:** Breast cancer, Oncology, Exercise, Strength training, Endurance training, Fatigue, Sleep, Insomnia, Quality of life, Patient-reported outcomes

## Abstract

**Background:**

The aim of this secondary analysis of the BENEFIT randomized controlled trial was to investigate the effects of aerobic training (AT) or resistance training (RT) during neoadjuvant chemotherapy (NACT) on sleep and cancer-related fatigue (CRF), compared to a waitlist control group (WCG) that performed RT after surgery.

**Methods:**

In the BENEFIT study, 184 patients with breast cancer with scheduled NACT (mean age = 50 years, standard deviation = 11) were randomized to AT (*n* = 62), RT (*n* = 62), or WCG (*n* = 60). While the AT and RT groups trained during NACT (two supervised and one home-based session weekly), the WCG completed the same training as the RT group but only after breast surgery. Self-reported sleep quality (Pittsburgh Sleep Quality Index) and CRF (EORTC QLQ-FA12) were collected before NACT (T0), after 9 weeks (T1), after NACT and before surgery (T2), 6 months after surgery (T3), and 12 months after surgery (T4). At T0, T2, and T3, sleep was additionally objectively measured by actigraphy.

**Results:**

Longitudinal analyses of covariance examining changes from baseline suggested no clear difference of AT and RT compared to the WCG regarding sleep and CRF parameters post-intervention (T2). In contrast, at T3 the WCG, which exercised between T2 and T3, showed more favorable mean values compared to the AT group in total CRF (adjusted mean difference (AMD): − 10.53, 95% CI [− 19.63, − 1.42]) and physical CRF (AMD: − 14.28 [− 26.02, − 2.54] on 0–100 scale), and a tendency toward lower scores in self-reported global sleep quality (AMD: − 0.24 [− 0.48, 0.01] on log-transformed scale). Moderation analyses further suggested that group differences in total CRF at T3 in favor of the WCG were more pronounced among participants with at least mild emotional distress at baseline. There were no clear differences between groups in objective sleep parameters at T2 or T3, or regarding self-reported sleep or fatigue endpoints at T4.

**Conclusions:**

The findings suggest that exercise interventions in the post-NACT phase may be more effective than during NACT for managing fatigue, while providing limited benefits for sleep.

**Trial registration:**

The BENEFIT study has been registered at ClincialTrials.gov (NCT02999074).

**Supplementary Information:**

The online version contains supplementary material available at 10.1186/s12916-026-04669-3.

## Background

Breast cancer is the most prevalent cancer among women, accounting for 23.8% of all cancer cases worldwide, and 74,016 new cases in Germany alone in 2022 [1, 2]. In the treatment of breast cancer, neoadjuvant chemotherapy (NACT), administered prior to surgery, has become widely accepted [3, 4]. The American Society of Clinical Oncology (ASCO) guidelines recommend NACT for different tumor types (e.g., high-risk triple-negative or HER2-positive) or when tumor shrinkage may allow for less extensive surgery, e.g., with breast conservation [5].

Cancer-related fatigue (CRF) and sleep disturbances affect the majority of patients with cancer during their treatment [6, 7] and are among the most distressing symptoms they experience [8, 9]. Both symptoms have been found to worsen during adjuvant chemotherapy [10, 11], and recent findings also indicate a deterioration in CRF and sleep quality following NACT [12, 13]. Therefore, we believe that interventions during NACT that alleviate CRF and sleep disturbances have the potential to provide significant clinical benefit.

Exercise interventions have shown a high effect on CRF during adjuvant chemotherapy [14] and a small effect on self-reported sleep quality that was not moderated by treatment status [15]. Accordingly, the American College of Sports Medicine (ACSM) recommends at least 30 min of at least moderate-intensity aerobic exercise three times per week to effectively improve both symptoms, while resistance training may also be beneficial for managing fatigue [16].

However, there is limited evidence on the effects of exercise during NACT on CRF and sleep. Small feasibility studies suggest that exercise during NACT is feasible and may reduce CRF [17–20]. Furthermore, most studies on exercise and sleep or CRF have used aerobic exercise alone or in combination with resistance training, while only a few studies examined resistance training alone [14–16, 21].

Treatment with NACT offers the opportunity to begin an exercise intervention already before surgery, which may not only improve patients’ physical fitness for surgery through prehabilitation [22] but also mitigate the early onset or persistence of sleep disturbances and CRF. As no randomized controlled trial (RCT) has yet compared the effects of exercise training on sleep and CRF during NACT versus post-surgery, the optimal timing for an exercise intervention to best manage these symptoms remains undetermined.

Therefore, the aims of this secondary analysis of an RCT are a) to investigate the effects of two exercise interventions (aerobic or resistance training) during NACT on sleep and CRF, and b) to explore whether these exercise interventions during NACT result in better medium- or long-term effects on CRF and sleep compared to an exercise intervention (resistance training) after surgery.

## Methods

### Study design and participants

These are secondary analyses of the BENEFIT trial, a 3-arm randomized controlled trial investigating AT and RT in patients with breast cancer undergoing NACT [23]. The trial was registered during ongoing data collection before data analysis at ClinicalTrials.gov (NCT02999074). This trial is reported in accordance with the CONSORT 2010 guidelines (see completed CONSORT checklist in Additional File 1).

Ethical approval for the study was obtained from the Ethics Committee of the Medical Faculty of the University of Heidelberg (S-678/2015). All study participants provided informed consent prior to enrollment. Participants were recruited predominantly at the National Center for Tumor Diseases (NCT) Heidelberg from January 2016 to October 2022. Few participants from external clinics who were able and interested to follow the study procedures were also recruited, as well as two participants at a cooperating center at University Medical Center Utrecht (The Netherlands), where the same training interventions and assessments (using translated questionnaires) were performed.

Eligibility criteria and details of recruitment procedures and assessments have been described previously [24]. Briefly, eligible study participants were adult female patients with breast cancer scheduled for (but not yet started) NACT, with a body mass index ≥ 18 kg/m^2^, sufficient language skills, willingness to participate in the study measurements, and to exercise twice a week in one of the cooperating gyms. Individuals who were already exercising systematically (i.e., at least 2 × 1 h/week) or had any contraindications for resistance or high-intensity aerobic training (e.g., acute infectious disease, severe cardiac disease, severe respiratory insufficiency) were not included. Participants were not specifically screened for sleep disturbances or CRF as an inclusion criterion. Eligible participants were randomized following a 1:1:1 ratio to an aerobic exercise training (AT), to a resistance exercise training (RT), both during NACT, or to a waitlist control group (WCG). A blocked randomization with randomly varying block size and stratified by tumor type (HR −; HR + and HER2 +; HR + and HER2 −) was performed using a computer-generated randomization list. A biometrician, who was not involved in the recruitment of the participants, performed the allocation after completion of the baseline assessments based on the computer-generated list. Other study personnel, including those who enrolled participants, had no access to the randomization list and no knowledge about block sizes to ensure allocation concealment. Blinding of participants for group allocation is not feasible in exercise intervention studies.

**Fig. 1:**
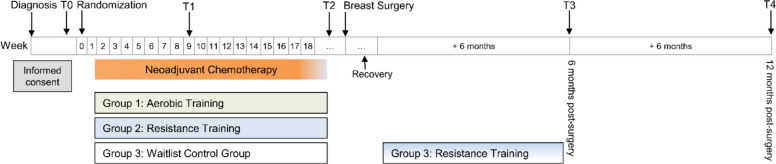
Study design of the BENEFIT trial. *Note:* The duration of the interventions during neoadjuvant chemotherapy varied according to the individual length of chemotherapy treatment (aerobic training group: *M* = 18.3 weeks, *SD* = 4.3; resistance training group: *M* = 17.8 weeks, *SD* = 4.3), while the resistance training for the waitlist control group was fixed at 18 weeks

Study participants completed paper-based questionnaires and further assessments at five time points: before the start of NACT (T0, baseline), 9 weeks after the start of NACT as a mid-intervention time point (T1; not used for inferential analyses), after the completion of NACT and before surgery (T2), as well as follow-up assessments at 6 months (T3) and 12 months (T4) after breast surgery (see Fig. 1). Decisions regarding the NACT regimen were made entirely as part of routine clinical care, without any involvement of the BENEFIT study personnel.

### Interventions

All groups exercised twice weekly at NCT Heidelberg/Heidelberg University Hospital facilities or at specially certified training facilities near to the participants’ homes (network OnkoAktiv; https://netzwerk-onkoaktiv.de/). These facilities were staffed with oncologically trained personnel who supervised the training sessions and were trained by study staff to ensure correct implementation of the protocol. After an individual 1:1 introduction session, supervision was provided in small group settings with at least one professional being present on the training floor at all times supporting participants following their exercise program. Participants received standardized instructions from the study team on how to perform their exercises and how to progressively increase the intensity. Both programs followed the ACSM exercise guidelines for survivors of cancer [16], applying the commonly recommended intensity range of 60–80% VO₂max (except for the high intensity interval training in the AT group). The programs lasted for the AT and RT groups throughout the full course of NACT until T2 assessment, which was a median (Q1; Q3) of 22 (18; 28) days after the last NACT infusion and always before surgery. Thus, the duration of the intervention period was aligned with the length of the individual chemotherapy regimens and varied slightly between groups (AT: mean (*M*) = 18.3 weeks; standard deviation (*SD*) = 4.3); RT: *M* = 17.8 weeks; *SD* = 4.3).

#### Aerobic training

Participants in the AT group began with 6 weeks of endurance training, usually on a cycle ergometer, at 60% of their maximal oxygen uptake (VO₂max). This was determined by baseline cardiopulmonary exercise testing until voluntary exhaustion. During training, adherence to the prescribed intensity was monitored via heart rate, watt output on ergometers, and the rating of perceived exertion (RPE) scale [25], which served as practical proxies for the VO₂max-derived targets. Participants were instructed to perform a short warm-up (around 5 min) at low intensity before each session. The training duration started with at least 15 min and was prolonged by 3–5 min in every exercise session based on RPE rating and the trainer’s decision so that the participant was able to perform the training session for 30 min at her 60% VO_2_max at the end of the second exercise week. Beginning with the third exercise week, the duration of one exercise session was gradually increased (see above) to 60 min (if possible) at an intensity of 70% VO_2_max. From week 7 to week 18 (or the end of NACT, respectively), participants performed a structured interval training consisting of four 4-min intervals at 75–85% VO_2_max, separated by 3-min recovery intervals at 60% VO_2_max.

#### Resistance training

Participants in the RT group performed a machine-based strength training involving all major upper and lower muscle groups. After two habituation sessions, participants completed a one-repetition maximum (1-RM) test on the following machines: leg press, leg extension, leg curl, seated row, latissimus pull-down, internal and external shoulder rotation, butterfly, and reverse butterfly. The training weight began at 60% 1-RM and was gradually increased up to 80% based on participant ability. Three sets of 8–12 repetitions were performed per exercise with 1-min rest between sets. If participants completed 12 repetitions in all three sets for three consecutive sessions, the weight was increased by 5%. No subjective criteria were applied to determine progression.

#### Waitlist control group

Participants randomized to the WCG received no exercise intervention during NACT but started the RT program at least 6 weeks after their breast surgery (*M* = 8.4 weeks; *SD* = 1.9) following written medical clearance from their treating physician confirming the absence of post-operative or other health issues that would contraindicate RT. Their program followed the same protocol as described above for the RT group and was standardized to 18 weeks.

All participants were also instructed to additionally perform a 15-min home-based exercise regimen once weekly without supervision. The AT group was advised to perform aerobic exercises such as walking, jogging, or cycling depending on the participant’s ability and interests, whereas the RT and WCG groups were instructed to perform resistance-based core stability exercises without additional weights. These unsupervised sessions focused on body-weight exercises engaging core and upper body muscles (e.g., planks, bridges, push-ups, or similar stability-oriented movements). In addition to the structured exercise sessions, study personnel contacted participants approximately every 2 weeks by phone to monitor their well-being, promoted adherence to the training recommendations, and provided individualized motivational support. These phone calls were also used to check for any health issues. Additionally, participants completed a brief questionnaire before and after each exercise session to assess acute health status and to report any serious exercise-related adverse events. Session attendance was defined as the number of completed supervised sessions divided by the number of prescribed sessions during the intervention period. After completion of the interventions, participants were not systematically encouraged to continue exercising, but exercise maintenance was evaluated in a separate study [26].

### Measures

#### Subjective measures of sleep behavior

Sleep quality was assessed using the Pittsburgh Sleep Quality Index (PSQI) [27]. The PSQI contains 19 self-rated items representing seven components of sleep behavior: sleep quality, sleep latency, sleep duration, habitual sleep efficiency, sleep disturbances, use of sleep medications, and daytime dysfunction (answered on a Likert scale from 0 to 3). The components were summed according to the PSQI scoring guide [27] to produce a global sleep quality score ranging from 0 to 21. The PSQI is a well-established instrument that has shown satisfactory reliability and construct validity in patients with cancer [28, 29].

#### Sleep diary

Participants kept a diary for the entire time they wore the accelerometer (see below), recording the time they (a) intended to sleep (light off) and (b) got up (light on). We explained to participants how to complete the fields, e.g., not to report the time they went to bed, but the time they intended to go to sleep.

#### Objective measures of sleep behavior

To record sleep behavior, participants wore accelerometers (ActiGraph device wGT3X-BT) on their non-dominant wrist for seven consecutive days at T0, T2, and T3. The setup and initiation of the ActiGraphs were supported by study personnel, and their use was explained in detail to the participants during the on-site visit. ActiLife software version 6.13.4 was used to analyze the sleep data. These data were generated in 60-s epochs at a sampling rate of 32 Hz and scored using the Cole-Kripke algorithm [30]. Sleep time was calculated using the ActiLife algorithm [31], which is based on the Tudor-Locke algorithm [32]. All sleep periods were individually checked for plausibility. Only participants whose sleep measurement covered at least four nights at one measurement time were included (resulting in the exclusion of 9 assessments); sleep times during the day were not taken into account. The following sleep variables generated by the ActiLife software were used: total sleep time (TST; time in minutes between sleep onset and awakening), sleep efficiency (SE; percentage of bedtime spent sleeping), number and duration of awakenings, and wake after sleep onset (WASO). Sleep duration, as well as duration and number of awakenings, and WASO, summarized as “sleep quality” in the trial registration, were analyzed to obtain a more detailed understanding of the interventions’ effects on different aspects of sleep behavior.

For participants with sleep diary data available, these were used to determine the start and end of bedtime. The bedtime was chosen as the time with the smallest difference between the diary entry and an actigraphy-detected sleep period, and the wake-up time was chosen as the time with the smallest difference between the diary entry and an ActiGraph-detected wake-up time. In a previous study comparing the validity of subjectively and objectively assessed sleep parameters, we found that the ActiGraph often has problems correctly assessing sleep onset latency [33]. For this reason, we chose the time of intended sleep time in the diary as the time of bedtime for sleep onset latency assessment (participants without diary data were excluded for this analysis).

#### Cancer-related fatigue

As stated in the trial registration, we initially focused on the Fatigue Assessment Questionnaire (FAQ) [34] to measure CRF. However, we also included a 13-item instrument developed by the European Organisation for Research and Treatment of Cancer (EORTC), which was still in the evaluation phase at the time but was later internationally validated and released as the EORTC QLQ-FA12 [35]. As it became a widespread standard tool for CRF, we amended the CRF assessment accordingly. The EORTC QLQ-FA12 consists of 12 items and covers the physical, affective, and cognitive dimensions of CRF and has demonstrated sensitivity to change [36] and good reliability, while confirming the three-dimensional structure of CRF [35]. The CRF total and subscores were calculated by summing the responses (1 = “not at all” to 4 = “very much”) and transforming them into a 0 to 100 scale, following the EORTC QLQ-C30 scoring manual [37].

#### Emotional distress

The Patient-Health-Questionnaire-4 (PHQ-4) was used for the assessment of emotional distress, consisting of two items measuring depressive symptoms from the Patient-Health-Questionnaire-9 and two items measuring anxiety symptoms, each on 4-point Likert scales from (1 = “not at all” to 4 = “almost every day”) [38].

#### Sociodemographic and medical characteristics

Marital status, education, and occupation were self-reported by participants at T0. At baseline, the study personnel also measured participants’ height and weight, which were subsequently used to calculate the body mass index (BMI) in kg/m^2^. Information on the start and end of NACT, cytostatic drugs received, and tumor characteristics were derived from medical records. Information on adjuvant chemotherapy, radiotherapy, hormonal therapy, antibody therapy as well as additional breast surgeries conducted after (primary) breast surgery were assessed via questionnaires at T3.

### Statistical analysis

No prospective statistical analysis plan was defined for the secondary outcomes reported in this manuscript. Descriptive statistics were used to report characteristics of study participants at baseline and potential differences between dropouts and completers. Adjusted mean differences (AMD) between the groups in CRF dimensions and subjective and objective sleep parameters at T2 (post-intervention) were analyzed using analysis of covariance (ANCOVA). The dependent variable in these models was the outcome value at T2 while the baseline value (T0) and the stratification factor tumor type (HR −, HR +/HER2 +, or HR +/HER2 −) were included as covariates (see Additional File 2 for examples of the statistical code). Similarly, group differences in all outcome values at T3 and T4 were investigated using equivalent ANCOVA models. For T3, models were additionally adjusted for treatments received between T2 and T3; for T4, models were adjusted for treatments received after T2 (i.e., chemotherapy, radiotherapy, hormonal therapy). No adjustment was made for antibody therapy due to high overlap with tumor type. The assumptions of the used statistical tests were checked beforehand. Due to concerns about a skewed distribution of the residuals, several of the outcome variables and their respective baseline values were transformed using the natural logarithm to improve model fit, as indicated with the results.

Further analyses were performed to assess interactions between intervention groups and symptom burden at baseline regarding the global score of the PSQI and the total fatigue score of the EORTC QLQ-FA12 at T2 and T3. To this end, we used the same ANCOVA models as for the main analysis and added a group × moderator interaction (see Additional File 2). Effect modifications were further explored in terms of AMD between groups at the respective time point if the *p*-value of the interaction was ≤ 0.10. The moderators were defined by the following subgroups at baseline: (1) emotional distress assessed with the PHQ-4 (no: < 3 (*n* = 81); at least mild: ≥ 3 (*n* = 81)), (2) total fatigue assessed with the EORTC QLQ-FA12 (low: < 33.3 (*n* = 134); high: ≥ 33.3 (*n* = 43)) according to the 75th percentile of 40–59-year-old women in the EORTC QLQ-FA12 from the general German population [39], (3) by the PSQI global score (good: ≤ 5 (*n* = 71); poor: > 5 (*n* = 88)). Further analyses also examined moderation effects by NACT agents, i.e., whether the regimen included platin derivates, cyclophosphamide, or anthracyclines (the respective case numbers are listed below; taxanes were not considered here as all except for one participant received them).

Patterns of missing data were examined by comparing participants who provided data at T2 and T3 (i.e., completers) to those who were lost to follow-up or did not return questionnaires at the respective time point (i.e., non-completers). Moreover, to address the issue of missing values, we reran the analyses on self-reported sleep and fatigue variables at T2 and T3 using Multiple Imputation by Chained Equations (MICE). A total of *n* = 30 imputed datasets were created using the mice package in *R* [40]. Each imputation model included the same variables as its statistical basis model (i.e., outcome’s baseline value, randomized group, tumor type, and additionally post-surgery treatment for T3) and potentially relevant auxiliary variables (i.e., age, BMI, employment, education, relationship status, exercise at baseline, emotional distress at baseline, global sleep quality or total fatigue at baseline, NACT agents, duration of NACT, and session attendance); post-baseline predictors were only allowed to inform follow-up outcomes (no look-ahead). For T2, session attendance was used only in the AT and RT group. This yielded a complete analysis set of all 184 participants, with estimates pooled across imputations using Rubin’s rules [41], which average point estimates and account for both within- and between-imputation variability.

Several sensitivity analyses for all outcomes at T2 and T3 were performed by excluding participants with (1) severe sleep problems at baseline (> 8 on the PSQI global score), (2) reported regular use of sleeping pills at the respective time point (i.e., value of 3 in the sleep medication component of the PSQI), (3) high total fatigue at baseline (> 33.3 on the EORTC QLQ-FA12, see above), (4) at least moderate emotional distress at baseline (≥ 6 on the PHQ-4), and (5) low participation in the exercise intervention (< 5 sessions attended).

All analyses took into account all available data at each time point, ensuring that all participants with at least one valid outcome measurement were included. Consequently, the sample size varies between time points. All statistical analyses were performed using R (version 4.5.1). The study was powered with respect to the primary endpoint of the study (tumor size). While statistical tests were conducted using a two-tailed approach with predefined significance thresholds (α = 0.05, and α = 0.1 for exploratory subgroup analyses), we did not report *p*-values, as this study was not powered for the secondary outcomes analyzed. Instead, we focused on mean differences and 95% confidence intervals to provide a more informative representation of the results. Pairwise post-hoc comparisons between intervention groups were Bonferroni-adjusted, but no global adjustments for multiple testing across outcomes or time points were applied.

## Results

### Study population

**Fig. 2:**
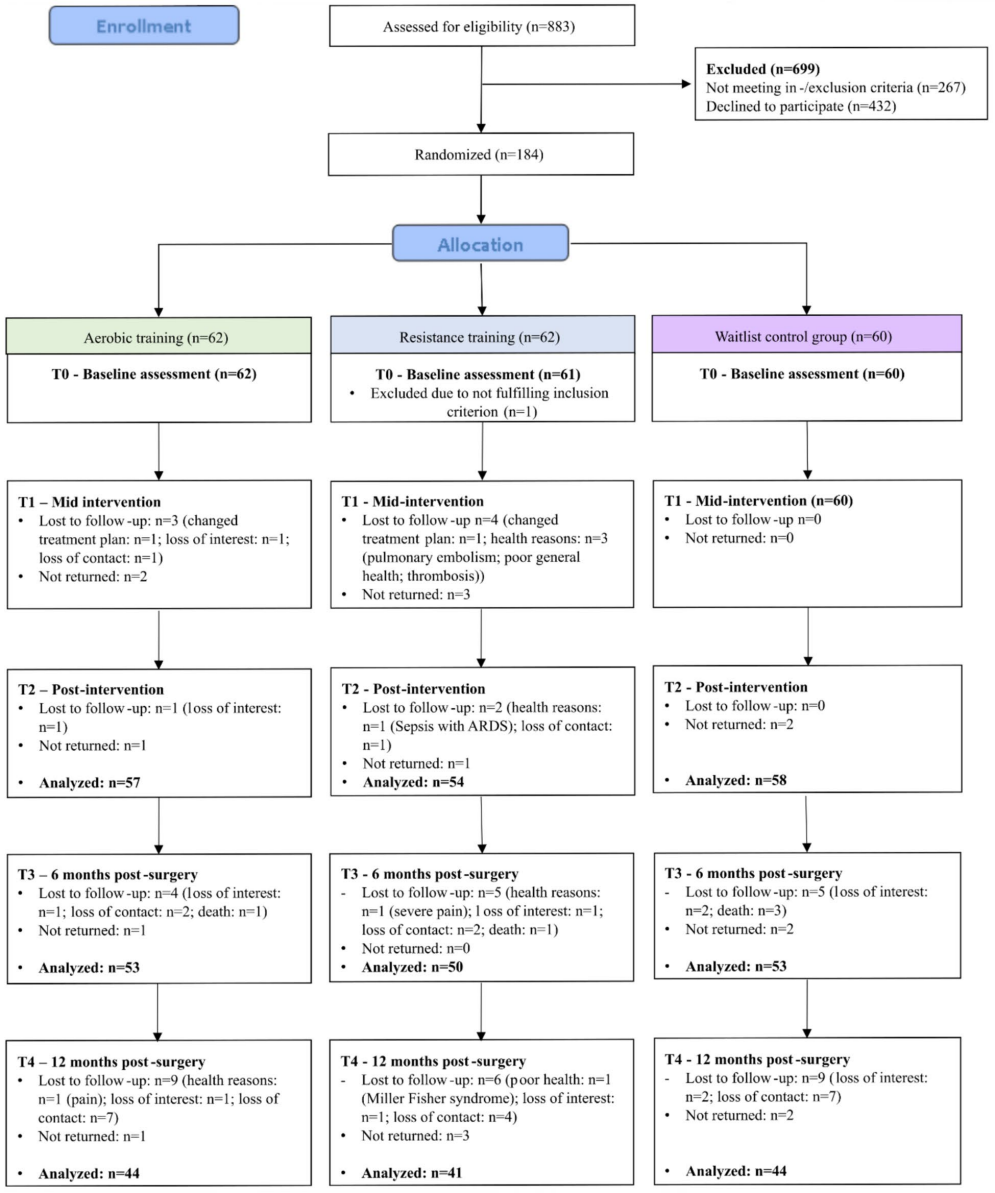
Study flow of participants through the trial. *Note:* Randomization took place after the baseline assessment but is shown differently here for presentation purposes; lost to follow-up: participants who discontinued study participation before the respective assessment for various reasons as self-reported by the participants; not returned: participants who remained in the study but did not return questionnaires for the respective assessment. *Abbreviation:* ARDS = acute respiratory distress syndrome

Of the 883 screened patients with breast cancer, *n* = 267 were not eligible due to inclusion or exclusion criteria and *n* = 432 declined to participate. Thus, 184 participants were randomized (62 = AT, 62 = RT, 60 = WGC). The entire study flow, including reasons for dropout from baseline to T4, is shown in Fig. 2. As actigraphy was added to the protocol after study start, objective sleep data were available for only *n* = 125 participants at T0 (100% with diary data), *n* = 117 at T2 (85.5% with diary data), and *n* = 110 at T3 (87.3% with diary data).

Participant characteristics by intervention group are described in Table 1. All three groups combined had a mean age of 50 years (*SD* = 11), a mean BMI of 26.3 kg/m^2^ (*SD* = 5.4), 79.2% were married or living with a partner, 54.6% had a diploma qualifying for university, and 27.9% were employed. Most common NACT agents were taxanes (*n* = 183, 99.5%), cyclophosphamides (*n* = 126, 68.5%), anthracyclines (mainly epirubicin; *n* = 116, 63.0%), and platin derivatives (*n* = 91, 49.5%). NACT was most frequently (34%) scheduled according to the schemes 4 × epirubicin/cyclophosphamide (90/600 mg/m^2^) followed by 12 × paclitaxel (80 mg/m^2^) or (nab-)paclitaxel (125 mg/m^2^) or vice versa; a further 22% received the scheme 4 × epirubicin/cyclophosphamide followed by 12 × (nab-)paclitaxel with the addition of carboplatin (AUC6). Another common NACT scheme (27%) was 6 × docetaxel/carboplatin (75 mg/m^2^/AUC6). A total of *n* = 72 (39.1%) study participants received additional antibody therapy, mainly trastuzumab. After surgery (thus, after T2), *n* = 26 (14.1%) received (adjuvant) chemotherapy*, n* = 103 (56.0%) received radiation therapy, *n* = 77 (41.8%) received hormonal therapy, *n* = 53 (28.8%) received antibody therapy, and *n* = 13 (7%) underwent at least one additional surgery. Analyses on exercise attendance rates in the AT and RT groups have been reported in detail elsewhere [23]. In this study population, participants in the AT group attended 41.6% of the sessions (*M *of sessions = 18.4; *SD* = 13.0), participants in the RT group attended 40.6% of the sessions (*M* = 16.3; *SD* = 12.1), and participants in the WCG attended 25.8% of the sessions (*M* = 11.6; *SD* = 11.7). There were no exercise-related serious adverse events.

**Table 1:** Sociodemographic and treatment-related characteristics of study participants

Variable	AT (*N* = 62)	RT (*N* = 62)	WCG (*N* = 60)
Age, mean (*SD*)	51 (12)	49 (11)	50 (10)
Body mass index, mean (*SD*)	25.8 (5.9)	25.7 (4.0)	27.6 (6.0)
Marital status, *n* (%)			
Married/living with a partner	48 (77.4)	49 (81.7)	48 (80.0)
Divorced/separated/single/other	14 (22.6)	11 (18.3)	12 (20.0)
Occupation, *n* (%)			
Currently employed	19 (30.6)	18 (29.5)	14 (23.3)
Not employed	43 (69.4)	43 (70.5)	46 (76.7)
Education, *n* (%)^a^			
Higher	34 (54.8)	29 (47.5)	35 (58.3)
Lower	28 (45.2)	32 (52.5)	25 (41.7)
Exercise [median MET-h/week, Q1–Q3] 12 months prior baseline	0.83 [0.0–4.0]	1.1 [0.0–3.9]	0.0 [0.0–2.7]
Emotional distress, mean (*SD*)^b^	54.6 (24.3)	52.5 (23.48)	50.0 (27.6)
Tumor type, *n* (%)			
HR +/Her2 +	13 (21.0)	15 (24.2)	11 (18.3)
HR +/Her2 −	26 (41.9)	25 (40.3)	23 (38.3)
HR −/Her2 +	5 (8.1)	3 (4.8)	2 (3.3)
HR −/Her2 −	18 (29.0)	19 (30.6)	24 (40.0)
Neoadjuvant therapy^c^, *n* (%)			
A + C + T + P	14 (22.6)	12 (19.4)	14 (23.3)
A + C + T	21 (33.9)	26 (41.9)	26 (43.3)
T + P	18 (29.0)	17 (27.4)	15 (25.0)
Duration^d^ [weeks], mean (*SD*)	18.3 (4.3)	17.8 (4.3)	17.6 (4.8)
Her2-targeting antibodies	18 (29.0)	18 (29.0)	13 (21.7)
Other antibodies	5 (8.1)	8 (12.9)	11 (18.3)
Adjuvant therapy, *n* (%)			
Chemotherapy	8 (12.9)	9 (14.5)	9 (15.0)
Radiation therapy	32 (51.6)	36 (58.1)	35 (58.3)
Hormonal therapy	28 (45.2)	25 (40.3)	24 (40.0)
Antibody therapy^e^	18 (29.0)	20 (32.3)	15 (25.0)
Additional surgery	2 (3.2)	6 (9.7)	5 (8.3)

*A *anthracyclines, *AT *aerobic training group, *C *cyclophosphamides, *MET-h/week *metabolic equivalent of task in hours per week, *P *platin derivates, *Q1 *first quartile, *Q3 *third quartile, *RT *resistance training group, *SD *standard deviation, *T *taxanes, *WCG *waitlist control group

^a^Higher: diploma qualifying for university or university degree; Lower: no degree or (lower-) secondary education degree

^b^Based on scores in EORTC QLQ-C30, 0–100 scale, of *n* = 176 participants (AT = 60; RT = 60; CG = 60)

^c^Only the most frequent combinations are mentioned here; others were T + C (*n* = 12), A + C (*n* = 2), A + T (*n* = 1), T (*n* = 6), T + P + A (*n* = 1)

^d^Time between first and last infusion

^e^Including Her2-targeting antibodies and other antibody therapies

The distributions of the self-reported raw subjective and objective sleep variables as well as CRF dimensions by group and time are presented in Additional File 3: Fig. S3a, Fig. S3b, and Fig. S3c.

### Group differences at post-intervention

Table 2 shows the differences between the groups at post-intervention (T2). The ANCOVA suggested no clear between-group differences (all confidence intervals included zero) between the groups in any of the sleep parameters assessed by the PSQI at post-intervention (T2) or in any of the CRF outcomes (i.e., total CRF, physical CRF, emotional CRF, or cognitive CRF). No meaningful between-group differences were observed in objective sleep parameters at T2 (see Additional File 4: Table S4a).

**Table 2:** Group differences between aerobic training (AT) and resistance training (RT) before surgery and resistance training after surgery (WCG) in sleep and fatigue parameters at post-intervention (T2)

Outcome	Range	Group	*N*	T0: Baseline	T2: Post-intervention	Overall group effect	AT vs. RT	AT vs. WCG	RT vs. WCG
				Mean^a^ (SD)	Mean^a^ (SD)	*F*(df1, df2), *p*	Mean difference [95% CI]^b^	Mean difference [95% CI]^b^	Mean difference [95% CI]^b^
PSQI: Global sleep score	0–21					*F*(2, 129) = 0.02, *p* =.978	0.01 [− 1.57, 1.60]	− 0.11 [− 1.68, 1.46]	− 0.12 [− 1.68, 1.43]
		AT	44	5.28 (3.00)	6.10 (3.17)				
		RT	44	6.45 (3.45)	6.61 (3.31)				
		WCG	47	6.57 (3.41)	6.78 (3.74)				
PSQI subscales									
Sleep quality	0–3					*F*(2, 154) = 0.88, *p* =.417	0.07 [− 0.23, 0.38]	0.17 [− 0.14, 0.47]	0.10 [− 0.20, 0.39]
		AT	52	1.01 (0.69)	1.28 (0.77)				
		RT	52	1.25 (0.62)	1.33 (0.65)				
		WCG	56	1.41 (0.65)	1.31 (0.71)				
Sleep latency	0–3					*F*(2, 143) = 0.15, *p* =.863	0.08 [− 0.36, 0.53]	0.09 [− 0.35, 0.52]	0.01 [− 0.43, 0.44]
		AT	49	0.90 (0.90)	1.18 (1.01)				
		RT	47	1.17 (1.03)	1.23 (1.01)				
		WCG	53	1.28 (1.08)	1.26 (1.00)				
Sleep duration^c^	0–3					*F*(2, 152) = 2.16, *p* =.119	− 0.07 [− 0.28, 0.13]	− 0.17 [− 0.37, 0.03]	− 0.09 [− 0.29, 0.10]
		AT	52	0.40 (0.44)	0.20 (0.40)				
		RT	50	0.47 (0.47)	0.30 (0.46)				
		WCG	56	0.45 (0.45)	0.39 (0.49)				
Sleep efficiency	0–3					*F*(2, 151) = 0.17, *p* =.845	− 0.11 [− 0.63, 0.41]	− 0.01 [− 0.52, 0.49]	0.10 [− 0.41, 0.61]
		AT	51	0.94 (1.07)	0.98 (1.14)				
		RT	50	1.10 (1.15)	1.16 (1.11)				
		WCG	56	1.02 (1.02)	1.02 (1.24)				
Sleep disturbances	0–3					*F*(2, 144) = 0.22, *p* =.806	0.06 [− 0.17, 0.30]	0.02 [− 0.22, 0.25]	− 0.05 [− 0.28, 0.19]
		AT	49	1.06 (0.52)	1.27 (0.49)				
		RT	49	1.13 (0.48)	1.22 (0.51)				
		WCG	52	1.19 (0.56)	1.29 (0.54)				
Daytime dysfunction	0–3					*F*(2, 153) = 0.49, *p* =.612	0.10 [− 0.20, 0.41]	0.11 [− 0.19, 0.41]	0.01 [− 0.29, 0.31]
		AT	51	0.71 (0.65)	1.22 (0.67)				
		RT	52	0.75 (0.62)	1.13 (0.60)				
		WCG	56	0.84 (0.60)	1.16 (0.71)				
Fatigue dimensions									
Total fatigue	0–100					*F*(2, 155) = 1.29, *p* =.277	0.61 [− 8.17, 9.39]	5.26 [− 3.52, 14.05]	4.65 [− 3.91, 13.21]
		AT	51	17.12 (15.67)	30.01 (18.51)				
		RT	53	21.27 (16.28)	31.73 (21.48)				
		WCG	57	25.68 (19.59)	29.71 (21.55)				
Physical fatigue	0–100					*F*(2, 155) = 0.85, *p* =.427	2.84 [− 9.20, 14.89]	6.48 [− 5.58, 18.54]	3.64 [− 8.11, 15.39]
		AT	51	21.50 (21.94)	45.49 (25.78)				
		RT	53	26.92 (20.84)	44.78 (25.80)				
		WCG	57	32.51 (24.98)	43.57 (27.75)				
Emotional fatigue	0–100					*F*(2, 155) = 0.52, *p* =.595	− 2.57 [− 13.60, 8.46]	1.95 [− 8.94, 12.85]	4.52 [− 6.22, 15.27]
		AT	51	22.66 (22.98)	21.79 (22.77)				
		RT	53	25.58 (24.13)	25.58 (28.21)				
		WCG	57	28.46 (28.95)	22.22 (24.67)				
Cognitive fatigue	0–100					*F*(2, 153) = 0.00, *p* =.999	− 0.13 [− 10.08, 9.82]	− 0.01 [− 9.76, 9.73]	0.12 [− 9.55, 9.79]
		AT	51	7.84 (15.40)	13.07 (19.81)				
		RT	52	12.82 (14.99)	16.19 (23.94)				
		WCG	56	11.01 (18.33)	15.18 (23.84)				

All values represent the subset of participants that were integrated in the respective analysis; No analyses were calculated for the Sleep Medication scale from the Pittsburgh Sleep Quality Inventory due to few cases (overall *n* = 14 with value > 0); *AT*, aerobic training group; *CI *confidence interval, *PSQI *Pittsburgh Sleep Quality Inventory, *RT *resistance training group, *SD *standard deviation, *WCG *waitlist control group

^a^Unadjusted mean values

^b^Group differences based on longitudinal analyses of covariance (ANCOVA) adjusted for baseline value of the outcome and tumor type (HR −, HER2 +/HR −, and HR +/HER2); post hoc comparisons between groups were adjusted using Bonferroni correction

^c^Variable was transformed to its natural logarithm

### Group differences at 6 months post-surgery

Table 3 shows the differences between the groups at 6 months after surgery (T3). Regarding the global PSQI score, there was a tendency towards a lower score, indicating better sleep quality, in the WCG (i.e., who had received the RT intervention between surgery and T3) compared to the AT group (AMD of − 0.24, 95% CI [− 0.48, 0.01]; on the log-transformed scale). The WCG also showed better sleep values with confidence intervals excluding zero compared to the AT group for the sleep latency subscale (AMD of − 0.47, 95% CI [− 0.92, − 0.02]) and compared to the RT group for the sleep quality subscale (AMD of − 0.35 [− 0.65 to − 0.04]). In addition, the WCG group had lower scores on total CRF (AMD of − 10.53 [− 19.64 to − 1.41]) and physical CRF (AMD of − 14.28 [− 26.03 to − 2.53]) compared to the AT group. There were no differences in objective sleep parameters between the groups at T3 (see Additional File 4: Table S4b).

**Table 3:** Group differences between resistance training after surgery (WCG) and aerobic training (AT) and resistance training (RT) before surgery in sleep and fatigue parameters at 6 months post-surgery (T3)

Outcome	Range	Group	*N*	T0: Baseline	T3: 6-month post-surgery	Overall group effect	AT vs. RT	WCG vs. AT	WCG vs. RT
				Mean^a^ (SD)	Mean^a^ (SD)	*F*(df1, df2), *p*	Mean difference [95% CI]^b^	Mean difference [95% CI]^b^	Mean difference [95% CI]^b^
PSQI: Global sleep score^c^	0–21					*F*(2, 120) = 2.79, *p* =.066	0.08 [− 0.16, 0.32]	− 0.24 [− 0.48, 0.01]	− 0.16 [− 0.40, 0.09]
		AT	43	1.79 (0.51)	1.95 (0.50)				
		RT	45	1.85 (0.54)	1.91 (0.54)				
		WCG	41	1.96 (0.58)	1.81 (0.59)				
PSQI subscales									
Sleep quality	0–3					*F*(2, 139) = 3.75, *p* =.026	− 0.14 [− 0.45, 0.17]	− 0.21 [− 0.52, 0.11]	** − 0.35 [− 0.65, − 0.04]**
		AT	50	1.08 (0.67)	1.22 (0.74)				
		RT	49	1.22 (0.62)	1.45 (0.79)				
		WCG	49	1.43 (0.67)	1.21 (0.65)				
Sleep latency	0–3					*F*(2, 134) = 3.67, *p* =.028	0.39 [− 0.06, 0.85]	** − 0.47 [− 0.92, − 0.02]**	− 0.09 [− 0.53, 0.37]
		AT	48	0.94 (0.86)	1.46 (1.05)				
		RT	46	1.11 (0.99)	1.20 (1.07)				
		WCG	49	1.35 (1.09)	1.20 (1.02)				
Sleep duration^c^	0–3					*F*(2, 138) = 0.54, *p* =.584	0.03 [− 0.18, 0.25]	0.06 [− 0.15, 0.27]	0.09 [− 0.12, 0.30]
		AT	50	0.42 (0.42)	0.32 (0.47)				
		RT	47	0.45 (0.46)	0.32 (0.44)				
		WCG	50	0.48 (0.46)	0.42 (0.49)				
Sleep efficiency	0–3					*F*(2, 137) = 0.68, *p* =.509	0.14 [− 0.37, 0.65]	− 0.24 [− 0.74, 0.26]	− 0.10 [− 0.60, 0.41]
		AT	50	1.06 (1.02)	1.16 (1.10)				
		RT	47	1.04 (1.14)	1.06 (1.07)				
		WCG	49	1.12 (1.03)	0.98 (1.16)				
Sleep disturbances	0–3					*F*(2, 135) = 0.34, *p* =.713	0.02 [− 0.23, 0.28]	− 0.08 [− 0.34, 0.17]	− 0.06 [− 0.31, 0.19]
		AT	48	1.08 (0.45)	1.29 (0.50)				
		RT	48	1.10 (0.43)	1.23 (0.52)				
		WCG	48	1.17 (0.52)	1.19 (0.57)				
Daytime dysfunction	0–3					*F*(2, 138) = 0.98, *p* =.377	0.02 [− 0.34, 0.38]	− 0.19 [− 0.55, 0.17]	− 0.17 [− 0.53, 0.19]
		AT	49	0.73 (0.67)	0.98 (0.78)				
		RT	49	0.73 (0.61)	0.94 (0.69)				
		WCG	49	0.84 (0.62)	0.84 (0.87)				
Fatigue dimensions									
Total fatigue	0–100					*F*(2, 139) = 4.24, *p* =.016	2.73 [− 6.39, 11.85]	** − 10.53 [− 19.64, − 1.41]**	− 7.80 [− 16.87, 1.27]
		AT	49	18.16 (15.73)	24.77 (18.14)				
		RT	48	20.19 (16.47)	23.50 (22.57)				
		WCG	51	26.91 (19.89)	20.10 (21.30)				
Physical fatigue	0–100					*F*(2, 139) = 4.57, *p* =.012	4.43 [− 7.32, 16.17]	** − 14.28 [− 26.03, − 2.53]**	− 9.85 [− 21.48, 1.78]
		AT	49	21.70 (21.21)	35.24 (23.77)				
		RT	48	26.25 (21.50)	33.06 (26.84)				
		WCG	51	33.59 (25.65)	27.19 (26.09)				
Emotional fatigue	0–100					*F*(2, 139) = 1.20, *p* =.304	0.12 [− 10.31, 10.56]	− 5.71 [− 15.96, 4.53]	− 5.59 [− 15.87, 4.69]
		AT	49	25.40 (24.22)	19.27 (22.20)				
		RT	48	24.31 (24.54)	19.68 (25.34)				
		WCG	51	30.07 (29.25)	16.56 (23.24)				
Cognitive fatigue	0–100					*F*(2, 138) = 0.61, *p* =.547	2.84 [− 6.00, 11.68]	− 3.78 [− 12.38, 4.81]	− 0.94 [− 9.59, 7.70]
		AT	49	9.52 (18.32)	11.90 (17.35)				
		RT	47	11.35 (14.79)	9.93 (20.46)				
		WCG	51	12.42 (18.81)	10.13 (18.88)				

All values represent the subset of participants that were integrated in the respective analysis; no analyses were calculated for the Sleep Medication scale from the Pittsburgh Sleep Quality Inventory due to few cases (overall *n* = 17 with value > 0); 95% confidence intervals that do not include 0 are marked in bold; *AT *aerobic training group, *CI *confidence interval, *PSQI *Pittsburgh Sleep Quality Inventory, *RT *resistance training group, *SD *standard deviation, *WCG *waitlist control group

^a^Unadjusted mean values

^b^Group differences based on analyses of covariance (ANCOVA) adjusted for baseline value of the outcome, tumor type (HR −, HER2 +/HR −, and HR +/HER2 −), and the treatment following the surgery until T3 (i.e., chemotherapy, radiotherapy, hormonal therapy); post hoc comparisons between groups were adjusted using Bonferroni Correction

^c^Variable was transformed to its natural logarithm

### Group differences at 12 months post-surgery

At 12 months postoperatively (T4), there were no differences (all confidence intervals included zero) between the three groups in any of the self-reported sleep parameters or CRF dimensions (see Additional File 5: Table S5).

### Analyses of subgroups

**Fig. 3:**
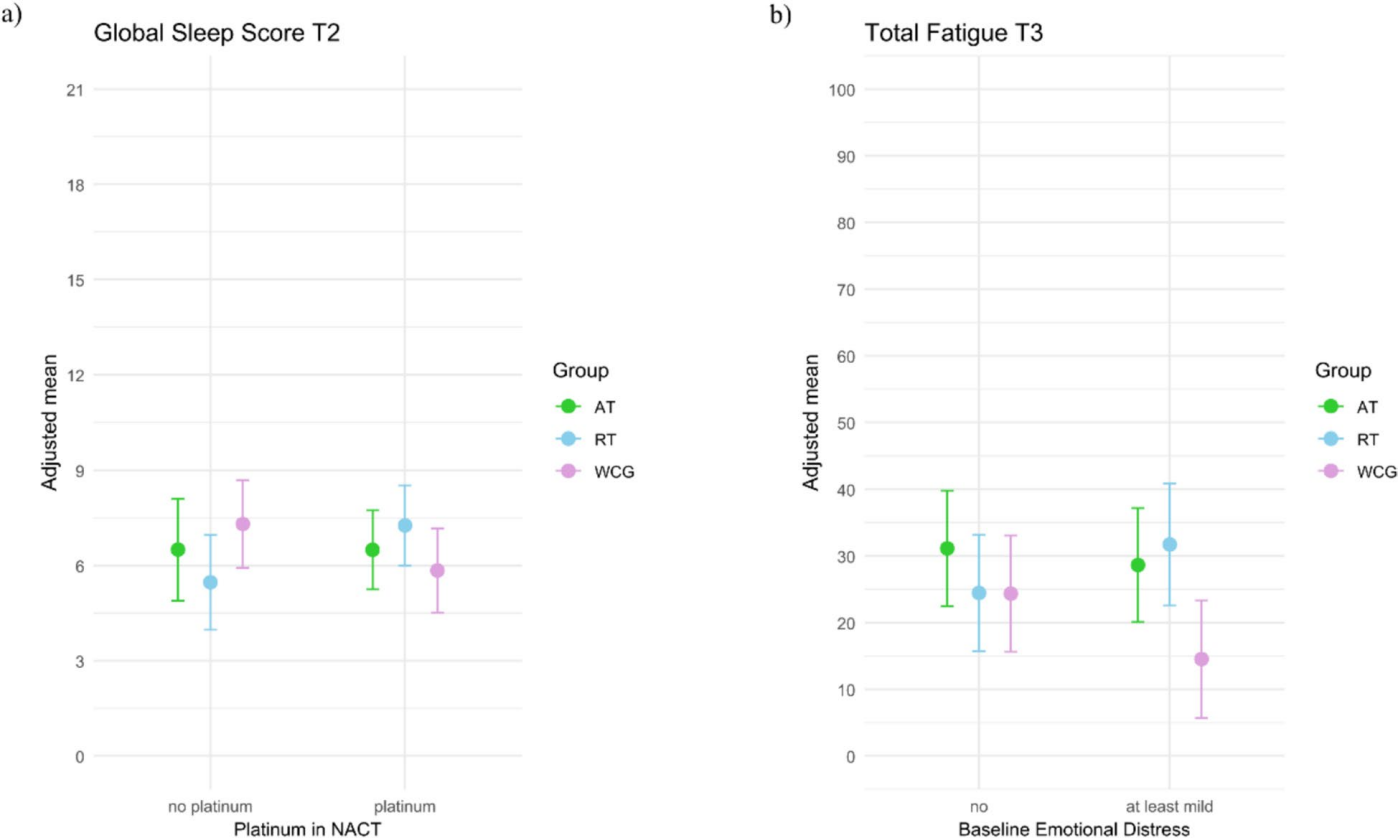
Subgroups differences in sleep and fatigue. **a** Adjusted global sleep score at T2 by whether the NACT regiment included platinum agent or not. **b** Adjusted total fatigue at T3 by baseline emotional distress. *Note:* Moderation analyses based on ANCOVAs were adjusted for the respective baseline value of the outcome and tumor type. For the T3 fatigue panel **b**, models were additionally adjusted for treatments received between T2 and T3 (chemotherapy, radiotherapy, hormonal therapy). Higher values indicate poorer global sleep quality and greater fatigue. Subgroups were defined as follows: baseline emotional distress by PHQ-4 (no: < 3; at least mild: ≥ 3); platinum in NACT: any platinum-based agent vs none. Error bars show 95% confidence intervals of adjusted means. *Abbreviations:* AT = aerobic training group; NACT = neoadjuvant chemotherapy; RT = resistance training group; WCG = waitlist control group

Exploratory analyses that examined moderations by symptom burden at baseline or on the use of specific NACT agents suggested two potential interaction effects on global sleep quality at T2 and total fatigue at T3 (see all interaction effects in Additional File 6: Table S6). First, we observed indications of an interaction between group and platinum on the global sleep quality at T2. Further analyses of AMD between groups showed that among participants whose NACT included platinum (WCG: *n* = 23; RT: *n* = 25), the adjusted mean global sleep score (higher scores indicating poorer sleep quality) tended to be higher in the RT group than in the WCG (*M* = 7.26 [6.00, 8.53] vs. *M* = 5.84 [4.52, 7.16]; AMD = − 1.42, 95% CI [− 3.54, 0.70]). In contrast, among those without platinum (WCG: *n* = 24; RT: *n* = 19), participants in the WCG tended to have a higher global sleep score than those in the RT group (*M* = 7.31 [5.92, 8.69] vs. *M* = 5.47 [3.97, 6.97]; AMD = 1.84, 95% CI [− 0.39, 4.07]). Second, explorative analyses suggested a potential interaction between group and emotional distress on total fatigue at T3; among participants with at least mild emotional distress at baseline (WCG: *n* = 26, RT: *n* = 9), total fatigue was lower in the WCG (*M* = 14.5, 95% CI [5.7, 23.3]) compared to participants in the RT group (*M* = 31.7, 95% CI [22.5, 40.9]; AMD = − 17.20, 95% CI [− 30.92, − 3.47]) (Fig. 3).

### Missing data analysis

#### Completer vs. non-completer comparisons

When comparing completers (*n* = 166) with non-completers (*n* = 16) at T2 using descriptive statistics, it became evident that non-completers at T2 were less likely to be married or living with a partner (56%) than completers (82%) and were more likely to have a HR +/HER2 − tumor status (65%) than completers (38%). Comparing completers (*n* = 156) and non-completers at T3 (*n* = 26), non-completers tended to be younger (*M* = 45.8; *SD* = 13.2) than completers (*M* = 50.5; *SD* = 10.4) and were less likely to have a higher educational degree (42%) than completers (56%). At T2, non-completers had slightly higher baseline symptom burdens in global sleep quality, total CRF, and emotional distress than completers, but differences were small (< 0.26 standardized mean difference (SMD)). A similar pattern was observed at T3, with non-completers showing slightly lower baseline symptom burdens with differences under 0.12 SMD.

#### Multiply imputed analyses

Analyses based on multiply imputed data that included all 184 randomized participants produced estimates that were highly consistent with the non-imputed results for self-reported sleep and fatigue, i.e., no differences between groups at T2 and advantages of the WCG regarding the global sleep score as well as total and physical fatigue at T3 (see Additional File 7: Table S7a and Table S7b).

### Sensitivity analysis

Sensitivity analyses did not show any differences regarding the results for the post-intervention (T2) outcomes. With regard to the group differences at 6 months after surgery (T3), excluding participants who attended fewer than five training sessions resulted in numerically larger differences in total fatigue between the WCG and both the RT group (AMD = − 11.56, 95% CI [− 21.53, − 1.59]) and the AT group (AMD = − 16.45, 95% CI [− 26.53, − 6.37]). With regard to emotional CRF, a notable difference between the WCG and AT group emerged at T3 (AMD = − 13.33, 95% CI [− 24.47, − 2.19]). We also observed lower levels of cognitive CRF in the WCG at T3 compared to the AT group when participants with clinically relevant CRF at baseline were excluded (AMD of − 6.32, 95% CI [− 13.20, 0.56]).

## Discussion

The BENEFIT RCT provides novel insights into the effects of AT and RT interventions during NACT on sleep and fatigue in patients with breast cancer. Inclusion of a WCG who received the resistance exercise intervention after completion of NACT and after surgery provides further insight into the effective timing of exercise interventions across the breast cancer continuum. In this secondary analysis, we did not observe indications of mitigating effects of either AT or RT intervention during NACT on sleep or fatigue parameters. In contrast, 6 months post-surgery, the WCG showed more favorable mean values in total and physical CRF and some self-reported sleep measures. Most other sleep parameters, including actigraphy-based sleep measures, showed no between-group differences. Differences between groups were no longer apparent at the 12-month post-surgery assessment.

While exercise during adjuvant chemotherapy has shown to improve CRF and sleep [14, 15, 42], we did not observe these effects during NACT. However, it may be premature to rule out the potential of exercise interventions to positively impact sleep and fatigue during NACT. First, both symptoms are based on a variety of causes and pathways [43, 44], which may lead to differential responses to exercise. Thus, future studies could further explore whether these underlying mechanisms influence exercise intervention outcomes during NACT. Second, our primary analyses estimated effects of assignment to AT or RT using all available data. With mean session attendance below 50%—which was also lower than reported in other exercise interventions during cancer treatment [18, 45–50]—participants received only a low exercise dose during NACT. Thus, clinically relevant per-protocol effects may have gone undetected. In addition, missing data further reduced the effective sample size for all outcomes, limiting statistical power and increasing uncertainty in the estimated effects. Our null findings should therefore be interpreted as the effect of offering exercise under real-world uptake during intensive therapy, rather than evidence that adequately dosed exercise is ineffective for sleep or CRF. Reasons for the low attendance rates in this study include COVID-19-related reluctance to go to the gym, selection of less physically active participants as inclusion criterion, and intensive treatment regimens during NACT [24]. To generally increase participation rates, additional professional support, particularly during intensive treatments [51, 52], or greater flexibility in session timing [45, 47] may be helpful. Since our study already included regular motivational support, further measures would likely be necessary in clinical practice to enhance attendance and effectiveness.

Third, high therapy burden itself may have contributed to the lack of beneficial effects of exercise on CRF and sleep post-NACT in our study. Notably, an individual patient data meta-analysis found no overall moderating effect of concurrent treatments on exercise effects on fatigue, although NACT regimens were not specifically examined [53]. In line with the German clinical guidelines [54], NACT regimens in our study population often included multiple taxane cycles and platinum-based chemotherapies, which are associated with various side effects [55]. In our exploratory moderation analyses, we observed an interaction with platinum-based therapy: receipt of a platinum agent was associated with poorer post-intervention sleep in the RT group (vs. WCG), whereas in participants not receiving platinum, the RT group tended to have better sleep quality than the WCG. We caution against over-interpretation, as subgroup sizes were small and confidence intervals for the adjusted mean differences were relatively wide; nevertheless, these findings suggest that treatment-related moderators such as platinum exposure merit closer examination in future studies. Likewise, antibody-based therapies such as HER2-targeted treatments, administered before and after surgery to about one in five participants in our cohort, are well known to be associated with fatigue and insomnia [56, 57]. Since different treatment regimens are associated with distinct side-effect profiles, they may influence symptom burden, adherence to oncological treatment as well as to exercise interventions, and the extent to which patients benefit from exercise. This underscores the importance of clarifying whether exercise interventions are most effective during treatment—when symptom burden is usually high—or afterwards to improve patient-reported outcomes. Interestingly, a study with a similar design, but not specifically focused on NACT, found that patients who exercised during chemotherapy reported better health-related quality of life, including less fatigue, immediately after treatment compared to a WCG that trained after chemotherapy [58]. Once the WCG had completed their intervention, the group differences disappeared, suggesting benefits of exercising both during and after treatment. The discrepancy with our study regarding effects during chemotherapy may be explained by the higher adherence to exercise interventions (≥ 75% in both groups) but also by the potentially lower treatment burden of adjuvant therapies compared to NACT regimens in the study population examined by van der Schoot [58]. Future research should examine the optimal timing of exercise interventions more closely.

At 6 months post-surgery, we observed substantial between-group differences in total and physical CRF in favor of the WCG who received the resistance exercise intervention after surgery compared to the groups who exercised during NACT. Notably, the advantage of the WCG at T3 for total and physical fatigue primarily reflects a stronger decline in CRF after NACT compared to the AT group, suggesting that post-surgical exercise may have supported fatigue recovery. The 10.5-point difference (that corresponds to a SMD of about 0.5) in total fatigue between WCG and AT exceeds effects reported in a recent meta-analysis of exercise interventions, such as aerobic exercise (SMD = 0.17) and resistance exercise (SMD = 0.37) [59]. It is also higher than the minimum clinically important difference of 8 points (assumed for the EORTC QLQ-C30 fatigue symptom scale) [60]. This clinically relevant advantage of the WCG in fatigue is particularly noteworthy, as mean session attendance in the WCG was only about 26%, even lower than in the groups that trained during NACT. In addition, the effect of the WCG was compared with groups that had previously received an exercise intervention, and where several participants continued the training or another type of exercise after completing the program (AT: 44.0%; RT: 55.2%; as previously published by Goldschmidt et al. [26]). Hence, the potential effect of the exercise intervention after surgery might be even underestimated. However, given the low attendance rate in the WCG and the continued exercise in parts of the AT and RT groups, actual differences in exercise exposure between groups were likely small. Thus, although the randomized design supports a causal interpretation, potential confounding factors cannot be fully ruled out and should be considered when interpreting these results. One possible confounding factor for the observed group differences at T3 could be baseline differences, with the WCG showing worse initial values in CRF and sleep parameters that may have regressed toward the mean over time. While our statistical models included baseline adjustment to reduce potential bias, some influence of these initial differences on the outcomes cannot be entirely excluded.

The fact that there was only a clear difference between the WCG and AT group, but not between WCG and RT, may not be over-interpreted, as the differences between WCG and RT pointed in a similar direction, and more participants of the RT group than of the AT group continued the training after surgery [26]. Nevertheless, future analyses of the different effects of RT versus AT on patient-reported outcomes are warranted. When considering different dimensions of CRF, our study showed, in agreement with previous literature, that the overall effect of exercise on CRF is mainly based on alleviation of physical CRF and less on emotional or cognitive CRF [50, 61]. To reduce cognitive and emotional CRF, additional psychosocial approaches, such as cognitive-behavioral therapy, which have also been shown to be effective in reducing CRF [62], may be helpful.

At 6 months after surgery, the WCG also reported better sleep quality, reflected by a trend toward lower global PSQI scores and clearly lower scores on the subscales sleep onset latency (vs. AT) and subjective sleep quality (vs. RT), while no clear differences were observed for the remaining PSQI subscales. In contrast to fatigue, the between-group differences in sleep outcomes at T3 appeared to be outcome-specific: while global sleep quality showed some improvement in the WCG, the other observed group differences were mainly driven by the worsening of these outcomes in the AT and RT groups after NACT. The adjusted mean difference of − 0.24 on the log-transformed scale of the PSQI global score corresponds to approximately 21% lower values in the WCG than in the AT group. Given the average baseline PSQI scores in our sample (around 6 points), this relative reduction would translate to less than 2 points on the original scale, thus well below the minimal clinically important difference of 4.4 points reported by Longo et al. [63], suggesting that the observed difference is unlikely to be clinically meaningful. Subjective improvements in sleep were not reflected in actigraphy-based parameters, a discrepancy also observed in previous studies [33, 42, 64]. Although diary-based sleep timing was used to enhance accuracy, biases in subjective perception of sleep quantity and quality and actigraphy limitations cannot be ruled out.

Subgroup analyses suggest that patients with higher baseline distress may respond differently to exercise. This aligns with previous studies showing greater benefits of exercise in patients with higher initial symptom burden [50, 65]. The results suggest that, in particular, patients who start therapy with at least mild emotional distress may show more favorable responses from exercise after surgery than during NACT. However, this finding should be interpreted cautiously due to the small sample sizes within the analyzed subgroups. Future research is needed to determine whether specific barriers or a higher need for psychosocial support—especially for those with high distress—exist in populations of patients with cancer undergoing NACT that may undermine the potential beneficial effects of an exercise intervention.

At 12 months after surgery, there were no longer any advantages of the WCG over the AT or RT group with respect to fatigue or sleep-related parameters. This is not surprising as we have already shown in previous analyses of this study that there were no differences between the three groups in voluntary exercise maintenance after the end of intervention [26]. This period often coincides with the conclusion of acute cancer treatment and the resumption of regular daily activities or working life. Given that the beneficial effects of exercise on QoL endpoints, including CRF, remain evident in cancer survivorship after treatment [66], it seems crucial to ensure adequate levels of exercise and physical activity in the long term.

The results of the study need to be considered with a number of limitations. As this is a secondary analysis, the study was not powered for the outcomes investigated here. We also did not adjust for multiple comparisons. Due to the large number of performed tests, none of the observed group differences would remain statistically significant after global correction for multiple testing across outcomes or time points. Thus, interpretation should not be guided by statistical significance but by the clinical relevance of mean differences. However, we acknowledge that multiple comparisons increase the risk of false-positive findings, and results should therefore be interpreted with caution. Furthermore, the generalizability of our findings may be limited, as the study was conducted only in patients with breast cancer, who were also eligible and willing to participate in a structured exercise program, which may introduce selection bias. Additionally, blinding of the participants was not possible due to the nature of the intervention and the self-reported outcomes. A major limitation of this study is the low adherence to the exercise interventions during NACT, which resulted in a substantially reduced delivered exercise dose. Consequently, the lack of effects on sleep and CRF during NACT may partly reflect insufficient exposure to the intervention rather than true inefficacy. This limitation is closely linked to a second major issue, namely substantial missing data, which further reduced the effective sample size and statistical power for all outcomes (e.g., PSQI global score: 27% missing at T2 and 30% at T3; EORTC QLQ-FA12 total fatigue: 13% at T2 and 20% at T3). To gauge potential bias, we compared completers and non-completers: While completers and non-completers showed slight sociodemographic and clinical differences, baseline symptom burden varied only marginally between completers and non-completers at both T2 and T3, suggesting limited selective-attrition bias. We also re-estimated the self-reported sleep and fatigue outcomes using multiple imputation, and pooled estimates closely matched the complete-case results, indicating robustness to missingness. Nonetheless, as multiple imputation assumes data are missing at random conditional on observed covariates, some residual bias cannot be ruled out.

A major strength of this study is the period of NACT, for which scientific evidence on the effects of exercise interventions is scarce. This study not only allowed investigations about which type of exercise (aerobic or resistance) may be beneficial compared to a control group during NACT but also provides insights into the timing of exercise intervention. This study further employed an elaborate assessment of endpoints, including both self-reported and objective measures (actigraphy) of sleep behavior and a three-dimensional assessment of cancer-related CRF. Additionally, the effects were investigated over an extended period, from before the start of NACT to 12 months post-surgery.

## Conclusions

Overall, while AT and RT during NACT had no effects on sleep or fatigue in patients with breast cancer, providing the resistance exercise intervention post-surgery yielded benefits. These were clinically meaningful for total and physical CRF, whereas group differences in sleep appear unlikely to be clinically relevant. Future studies should investigate how exercise adherence and psychosocial support may improve the effects of exercise interventions during NACT, particularly in patients with emotional distress. For patients unable or not willing to participate in a structured exercise program during NACT, interventions post-surgery may be more feasible and particularly beneficial for managing fatigue.

## Supplementary Information

Additional file 1. CONSORT 2010 checklist of information to include when reporting a randomised trial

Additional file 2. Statistical Code Examples

Additional file 3. Figures S3a-S3c. Figure S3a: Box-Whisker plots of raw self-reported sleep parameters across all measurement points. Figure S3b: Box-Whisker plots of objective sleep parameters across all measurement points. Figure S3c: Box-Whisker plots of raw self-reported fatigue parameters across all measurement points

Additional file 4. Table S4a-S4b. Table S4a: Group differences between aerobic trainingand resistance trainingbefore surgery and resistance training after surgeryin objective sleep parameters at post-intervention. Table S4b: Title: Group differences between resistance training after surgeryand aerobic trainingand resistance trainingbefore surgery in objective sleep parameters at 6 months post-surgery

Additional file 5. Table S5: Group differences between resistance training after surgeryand aerobic trainingand resistance trainingbefore surgery in sleep and fatigue parameters 12 months post-surgery

Additional file 6. Table S6: Moderation analyses: interaction effects on global sleep score and total fatigue at T2 and T3 by symptom burden at baseline and NACT agents

Additional file 7. Table S7a-S7b. Table S7a: Group differences between aerobic trainingand resistance trainingbefore surgery and resistance training after surgeryin sleep and fatigue parameters at post-interventionwith missing data imputation. Table S7b: Group differences between resistance training after surgeryand aerobic trainingand resistance trainingbefore surgery in sleep and fatigue parameters at 6 months post-surgerywith missing data imputation

## Data Availability

The datasets used and/or analyzed during the current study are available from the corresponding author on reasonable request.

## Abbreviations

ACSM: American College of Sports Medicine
AMD: Adjusted mean difference
ANCOVA: Analysis of covariance
ASCO: American Society of Clinical Oncology
AT: Aerobic exercise training
BMI: Body mass index
CRF: Cancer-related fatigue
EORTC: European Organisation for Research and Treatment of Cancer
FAQ: Fatigue Assessment Questionnaire
M: Mean
MICE: Multiple imputation by chained equations
NACT: Neoadjuvant chemotherapy
NCT: National Center for Tumor Diseases
PHQ-4: Patient Health Questionnaire-4
PSQI: Pittsburgh Sleep Quality Index
RCT: Randomized controlled trial
RPE: Rating of perceived exertion
RT: Resistance exercise training
SD: Standard deviation
SE: Sleep efficiency
TST: Total sleep time
VO₂max: Maximal oxygen uptake
WASO: Wake after sleep onset
WCG: Waitlist control group
1-RM: One-repetition maximum
