# Supplementary material for: Effects of aerobic or resistance exercise on sleep and cancer-related fatigue in patients with breast cancer during or after neoadjuvant chemotherapy: a 3-arm randomized controlled trial
Source: BMC Med. 2026 Jan 28;24:114. doi: 10.1186/s12916-026-04669-3 (PMC12924517; doi:10.1186/s12916-026-04669-3)
Supplement: Supplementary file 2 — Additional file 2. Statistical Code Examples [file 12916_2026_4669_MOESM2_ESM.docx]

**Statistical Code Examples**

To enhance transparency, we provide below example code snippets for the Analysis of Covariance with regard to the outcome total fatigue at different timepoints, implemented in R. All other models followed the same analytical logic, differing only in the specific outcome variables (and their respective baseline value).

**Main Analysis**

**A. T2 analysis:**

fit_t2 <- lm(

(eortc_fa_total.30 ~ group + eortc_fa_total.0 + tumtype, data = dataset)

emm <- emmeans::emmeans(fit, ~ group, cov.reduce = mean)

contr <- emmeans::contrast(emm, method =

list( "AT vs RT" = c(AT=1,RT=-1,WCG=0),

"AT vs WCG" = c(AT=1,RT=0,WCG=-1),

"RT vs WCG" = c(AT=0,RT=1,WCG=-1)),

adjust = "bonferroni")

**B. T3 analysis:**

fit_t3 <- lm(

(eortc_fa_total.30 ~ group + eortc_fa_total.0 + tumtype + chemo_t20_t30 + rad_t20_t30 + hormon_t20_t30, data = dataset)

emm <- emmeans::emmeans(fit, ~ group, cov.reduce = mean)

contr <- emmeans::contrast(emm, method =

list( "AT vs RT" = c(AT=1,RT=-1,WCG=0),

"WCG vs AT" = c(AT=-1,RT=0,WCG=1),

"WCG vs RT" = c(AT=0,RT=-1,WCG=1)),

adjust = "bonferroni")

**C. T4 analysis:**

fit_t4 <- lm(

(eortc_fa_total.30 ~ group + eortc_fa_total.0 + tumtype + chemo_after_op + radiation_after_op + hormone_after_op, data = dataset)

emm <- emmeans::emmeans(fit, ~ group, cov.reduce = mean)

contr <- emmeans::contrast(emm, method =

list( "AT vs RT" = c(AT=1,RT=-1,WCG=0),

"WCG vs AT" = c(AT=-1,RT=0,WCG=1),

"WCG vs RT" = c(AT=0,RT=-1,WCG=1)),

adjust = "bonferroni")

**Moderator Analysis**

1. **T2 analysis:**

fit_mod_t2 <- lm(

eortc_fa_total.20 ~ group * phq_score_cat.0 + eortc_fa_total.0 + tumtype,

data = dataset)

1. **T3 analysis:**

fit_mod_t3 <- lm(

eortc_fa_total.30 ~ group * phq_score_cat.0 + eortc_fa_total.0 +
tumtype + chemo_t20_t30 + rad_t20_t30 + hormon_t20_t30, data = dataset)

**Variable list**

- *eortc_fa_total.20/30/40*: Value of the total fatigue score at T2/T3/T4
- *group:* Randomized study arm
- *eortc_fa_total.0:* Baseline value (T0) of the total fatigue score
- *tumtype:* Tumor type (HR−, HR+ / HER2+, or HR+/HER2-)
- *chemo_t20_t30:* Dummy variable indicating chemotherapy between T2 and T3
- *rad_t20_t30:* Dummy variable indicating radiotherapy between T2 and T3
- *hormon_t20_t30:* Dummy variable indicating hormonal therapy between T2 and T3
- *chemo_after_op:* Dummy variable indicating chemotherapy after T2
- *radiation_after_op:* Dummy variable indicating radiotherapy after T2
- *hormone_after_op:* Dummy variable indicating hormonal therapy after T2
- *phq_score_cat.0:* Binary indicator of baseline emotional distress (PHQ-4 at T0): 0 = PHQ-4 < 3 (none), 1 = PHQ-4 ≥ 3 (at least mild)
