# Supplementary material for: Effects of aerobic or resistance exercise on sleep and cancer-related fatigue in patients with breast cancer during or after neoadjuvant chemotherapy: a 3-arm randomized controlled trial
Source: BMC Med. 2026 Jan 28;24:114. doi: 10.1186/s12916-026-04669-3 (PMC12924517; doi:10.1186/s12916-026-04669-3)
Supplement: Supplementary file 3 — Additional file 3. Figures S3a-S3c. Figure S3a: Box-Whisker plots of raw self-reported sleep parameters across all measurement points. Figure S3b: Box-Whisker plots of objective sleep parameters across all measurement points. Figure S3c: Box-Whisker plots of raw self-reported fatigue parameters across all measurement points [file 12916_2026_4669_MOESM3_ESM.docx]

| **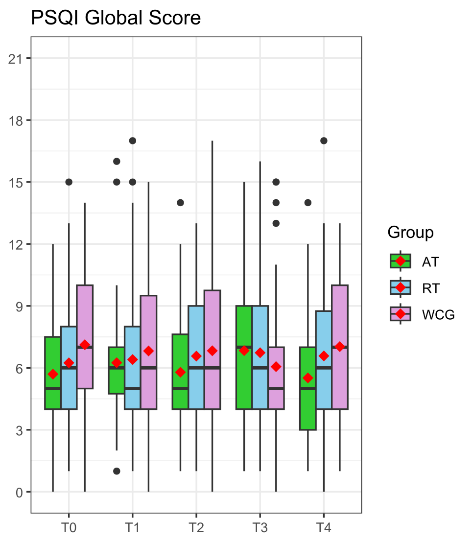** | **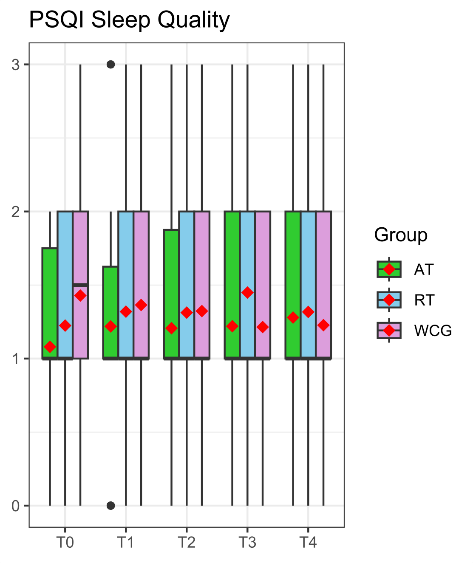** | | **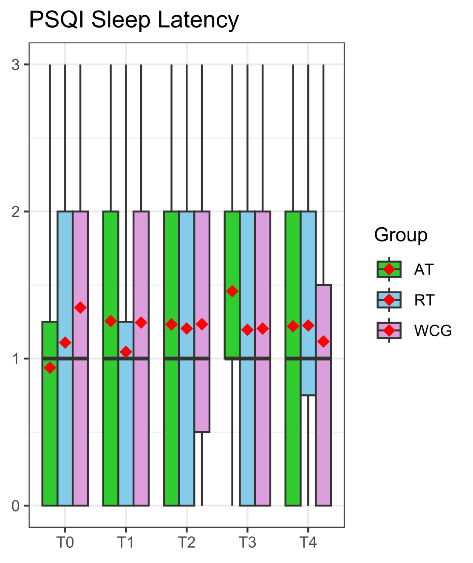** | | **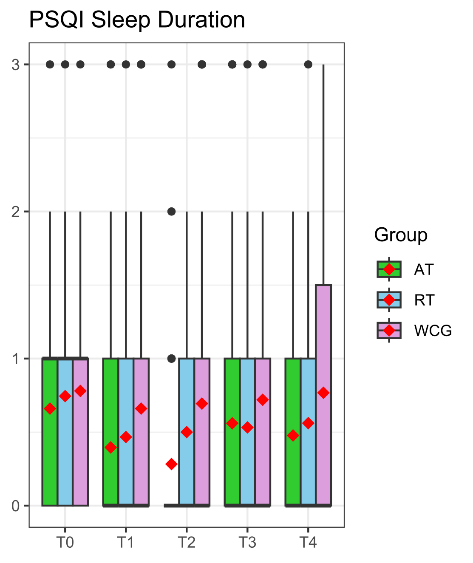** | |
| --- | --- | --- | --- | --- | --- | --- |
| **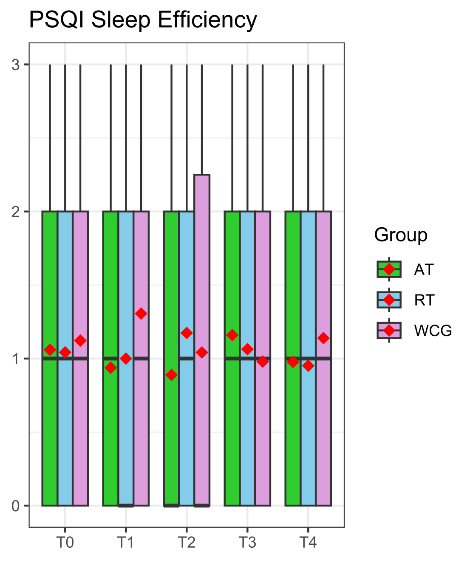** | | **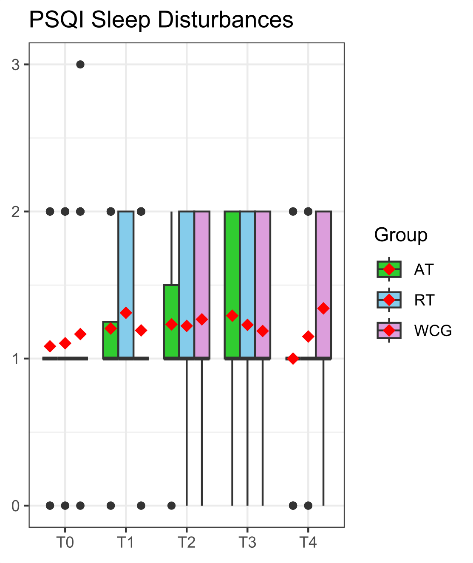** | | **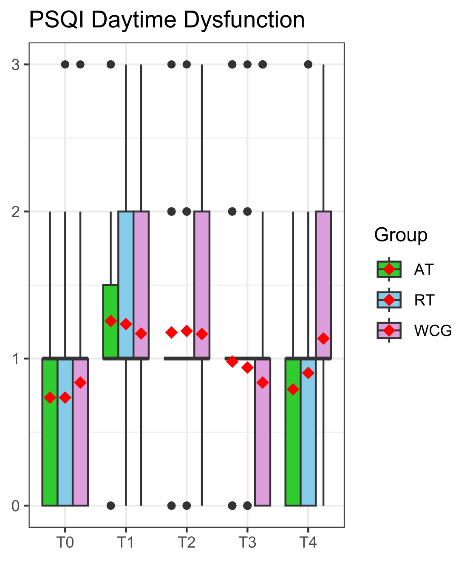** | |  |

**Figure S3a**. Box-Whisker plots of raw self-reported sleep parameters across all measurement points.

*Note:* Boxes represents 25th, 75th percentiles (interquartile range) with middle line in box at median; red dots represent mean values; the whiskers extended data points below or above 1.5 times the interquartile range; outliers (black dots) are outside this range; collapsed boxplots indicate zero interquartile range; AT: Aerobic Training Group; RT: Resistance Training Group; WCG: Waitlist Control Group; T0: Baseline; T1: 9 weeks after start of neoadjuvant chemotherapy (NACT), T2: After completion of NACT; T3: 6 months post-surgery; T4: 12 months post-surgery.

| **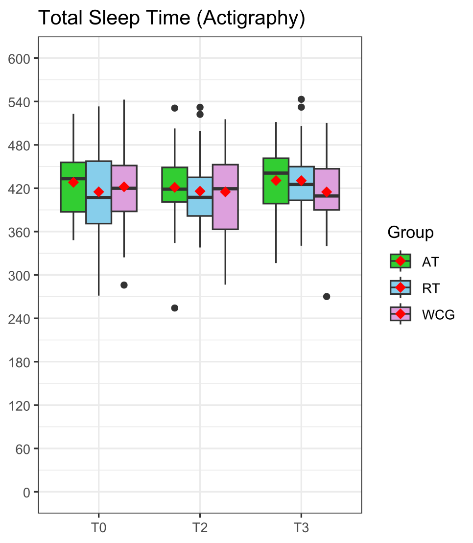** | **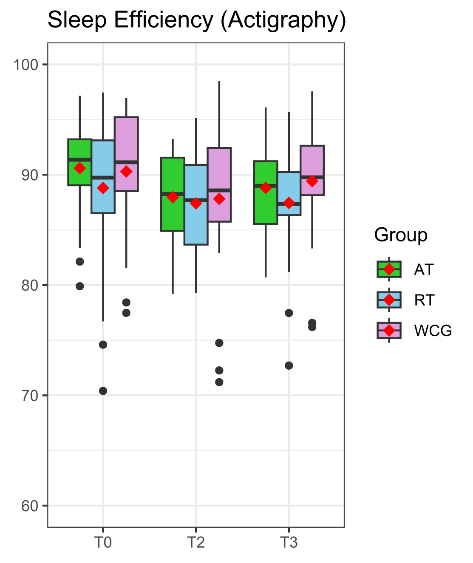** | **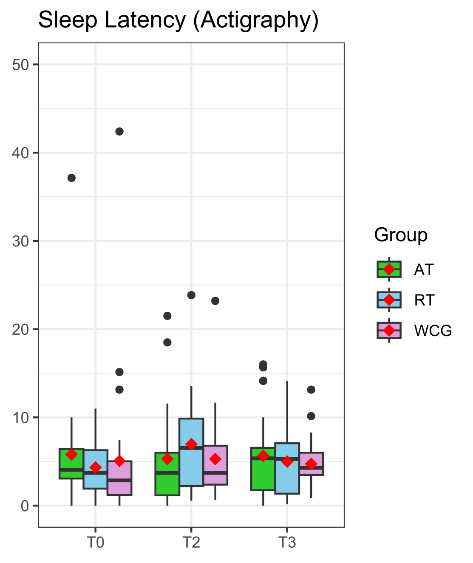** |
| --- | --- | --- |
| **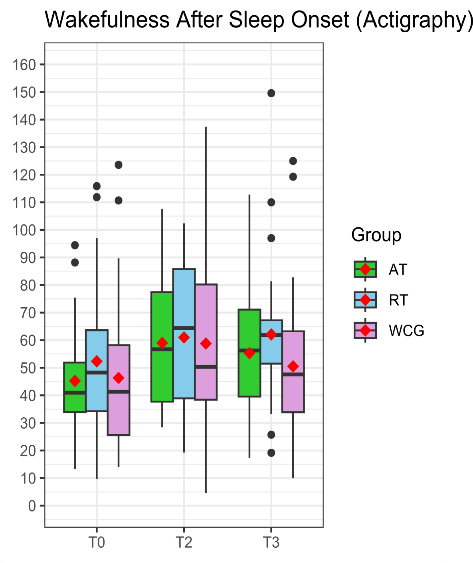** | **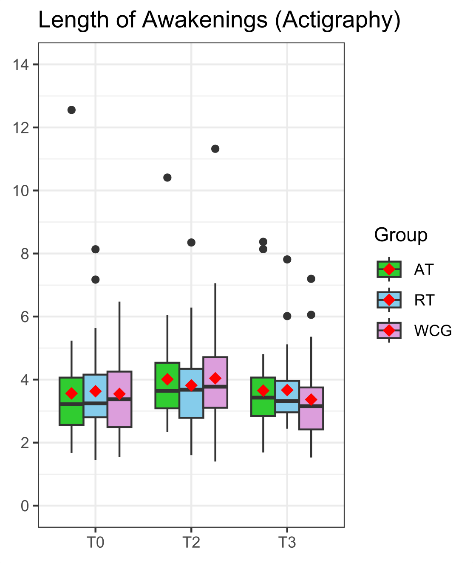** | **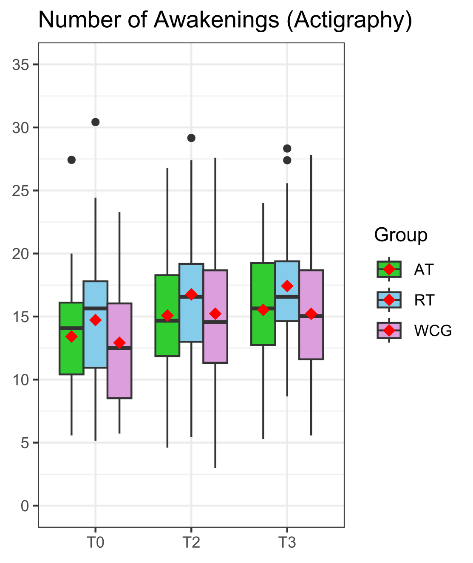** |

**Figure S3b**. Box-Whisker plots of objective sleep parameters across all measurement points.

*Note:* Boxes represents 25th, 75th percentiles (interquartile range) with middle line in box at median; red dots represent mean values; the whiskers extended data points below or above 1.5 times the interquartile range; outliers (black dots) are outside this range; AT: Aerobic Training Group; RT: Resistance Training Group; Waitlist Control Group; T0: Baseline; T2: After completion of NACT; T3: 6 months post-surgery.

| 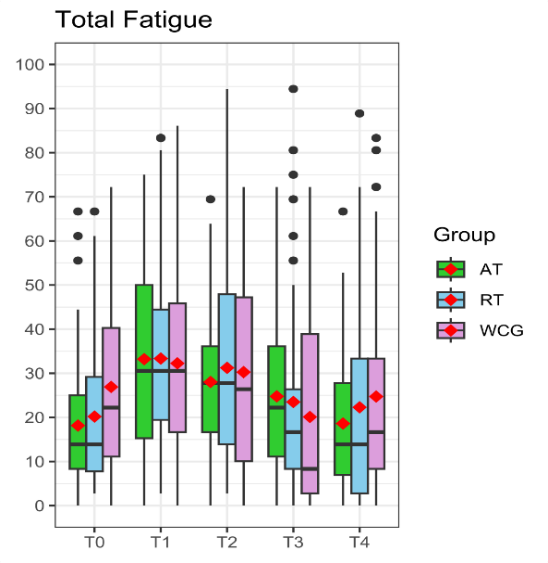 | 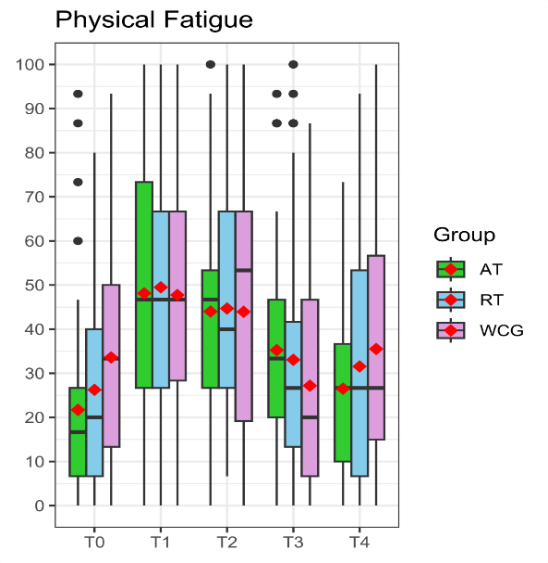 | **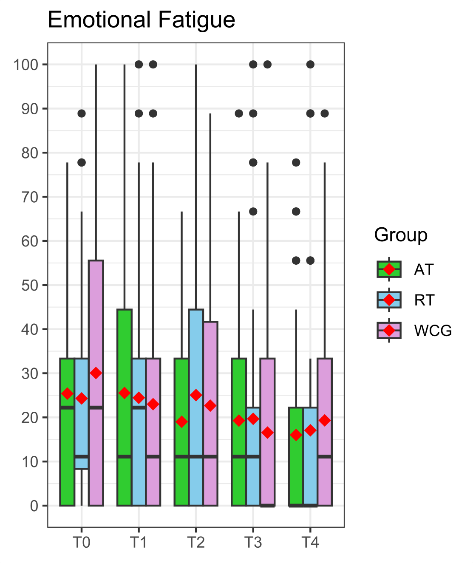** | **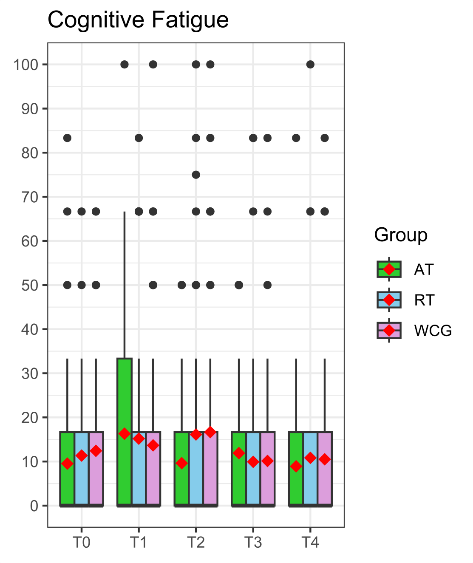** |
| --- | --- | --- | --- |

**Figure S3c**. Box-Whisker plots of raw self-reported fatigue parameters across all measurement points.

*Note:* Boxes represents 25th, 75th percentiles (interquartile range) with middle line in box at median; red dots represent mean values; the whiskers extended data points below or above 1.5 times the interquartile range; outliers (black dots) are outside this range; AT: Aerobic Training Group; RT: Resistance Training Group; WCG: Waitlist Control Group; T0: Baseline; T1: 9 weeks after start of neoadjuvant chemotherapy (NACT),+; T2: After completion of NACT; T3: 6 months post-surgery; T4: 12 months post-surgery.
