# Supplementary material for: Effects of aerobic or resistance exercise on sleep and cancer-related fatigue in patients with breast cancer during or after neoadjuvant chemotherapy: a 3-arm randomized controlled trial
Source: BMC Med. 2026 Jan 28;24:114. doi: 10.1186/s12916-026-04669-3 (PMC12924517; doi:10.1186/s12916-026-04669-3)
Supplement: Supplementary file 4 — Additional file 4. Table S4a-S4b. Table S4a: Group differences between aerobic trainingand resistance trainingbefore surgery and resistance training after surgeryin objective sleep parameters at post-intervention. Table S4b: Title: Group differences between resistance training after surgeryand aerobic trainingand resistance trainingbefore surgery in objective sleep parameters at 6 months post-surgery [file 12916_2026_4669_MOESM4_ESM.docx]

**Table S4a.** Group differences between aerobic training (AT) and resistance training (RT) before surgery and resistance training after surgery (WCG) in objective sleep parameters at post-intervention (T2)

| Outcome | Group | N | T0: Baseline | | T2: Post-Intervention | | Overall group  effect | AT vs. RT | AT vs. WCG | RT vs. WCG |
| --- | --- | --- | --- | --- | --- | --- | --- | --- | --- | --- |
|  |  |  | Mean^a^ | (SD) | Mean^a^ | (SD) | F(df1, df2), p | Mean difference [95% CI]^b^ | Mean difference [95% CI]^b^ | Mean difference [95% CI]^b^ |
| Total Sleep Time |  |  |  |  |  |  | F(2, 104) = 0.61, | 11.42 [-20.12, 42.97] | 12.21 [-17.21, 41.63] | 0.78 [-30.09, 31.66] |
|  | AT | 37 | 423.39 | (51.34) | 430.28 | (66.99) | p = .546 |  |  |  |
|  | RT | 32 | 416.85 | (60.86) | 417.67 | (48.53) |  |  |  |  |
|  | WCG | 41 | 425.42 | (48.35) | 420.39 | (54.57) |  |  |  |  |
| Sleep Efficiency^c^ |  |  |  |  |  |  | F(2, 104) = 0.07, | -0.00 [-0.03, 0.03] | 0.00 [-0.03, 0.03] | 0.00 [-0.03, 0.04] |
|  | AT | 37 | 4.51 | (0.08) | 4.48 | (0.05) | p = .929 |  |  |  |
|  | RT | 32 | 4.50 | (0.07) | 4.48 | (0.06) |  |  |  |  |
|  | WCG | 41 | 4.52 | (0.05) | 4.49 | (0.07) |  |  |  |  |
| Sleep Latency^c^ |  |  |  |  |  |  | F(2, 82) = 0.45, | -0.21 [-0.75, 0.33] | -0.11 [-0.63, 0.42] | 0.10 [-0.43, 0.64] |
|  | AT | 30 | 1.63 | (0.72) | 1.53 | (0.87) | p = .636 |  |  |  |
|  | RT | 29 | 1.40 | (0.71) | 1.68 | (0.77) |  |  |  |  |
|  | WCG | 29 | 1.49 | (0.73) | 1.59 | (0.93) |  |  |  |  |
| Wake after Sleep Onset |  |  |  |  |  |  | F(2, 104) = 0.13, p = .880 | 3.08 [-11.72, 17.87] | 1.55 [-12.28, 15.37] | -1.53 [-16.04, 12.99] |
|  | AT | 37 | 49.03 | (30.00) | 62.42 | (25.97) |  |  |  |  |
|  | RT | 32 | 51.66 | (27.34) | 60.14 | (26.50) |  |  |  |  |
|  | WCG | 41 | 45.16 | (24.69) | 58.66 | (32.40) |  |  |  |  |
| Number of Awakenings |  |  |  |  |  |  | F(2, 104) = 0.13, p = .880 | 0.63 [-2.45, 3.70] | 0.39 [-2.48, 3.27] | -0.23 [-3.25, 2.79] |
|  | AT | 37 | 14.04 | (5.58) | 16.58 | (6.51) |  |  |  |  |
|  | RT | 32 | 14.48 | (5.80) | 16.31 | (6.03) |  |  |  |  |
|  | WCG | 41 | 13.07 | (5.10) | 15.59 | (5.80) |  |  |  |  |
| Length of Awakenings |  |  |  |  |  |  | F(2, 104) = 0.15, p = .860 | 0.03 [-0.75, 0.82] | -0.13 [-0.85, 0.60] | -0.16 [-0.92, 0.60] |
|  | AT | 37 | 3.61 | (1.88) | 3.89 | (1.39) |  |  |  |  |
|  | RT | 32 | 3.61 | (1.48) | 3.81 | (1.39) |  |  |  |  |
|  | WCG | 41 | 3.47 | (1.32) | 3.93 | (1.83) |  |  |  |  |

*Note:* All values represent the subset of participants that were integrated in the respective analysis.

AT: Aerobic Training Group; CI: Confidence Interval; RT: Resistance Training Group; SD: Standard Deviation; WCG: Waitlist Control Group

^a^ Unadjusted mean values

^b^ Group differences based on analyses of coviarance (ANCOVA) adjusted for baseline value of the outcome and tumor type (HR-, HER2+/HR-, and HR+/HER2-); Post-hoc comparisons between groups were adjusted using Bonferroni Correction.

^c^ Variable was transformed to its natural logarithm

**Table S4b.** Group differences between resistance training after surgery (WCG) and aerobic training (AT) and resistance training (RT) before surgery in objective sleep parameters at 6 months post-surgery (T3)

| Outcome | Group | N | T0: Baseline | | T2: Post-Intervention | | Overall group  effect | AT vs. RT | WCG vs. AT | WCG vs. RT |
| --- | --- | --- | --- | --- | --- | --- | --- | --- | --- | --- |
|  |  |  | Mean^a^ | (SD) | Mean^a^ | (SD) | F(df1, df2), p | Mean difference [95% CI]^b^ | Mean difference [95% CI]^b^ | Mean difference [95% CI]^b^ |
| Total Sleep Time |  |  |  |  |  |  | F(2, 85) = 1.37, | -4.25 [-34.38, 25.87] | -13.82 [-42.62, 14.97] | -18.08 [-46.30, 10.14] |
|  | AT | 30 | 428.14 | (47.02) | 430.66 | (49.59) | p = .260 |  |  |  |
|  | RT | 30 | 415.00 | (61.96) | 430.44 | (47.37) |  |  |  |  |
|  | WCG | 34 | 421.97 | (52.99) | 414.85 | (48.87) |  |  |  |  |
| Sleep Efficiency |  |  |  |  |  |  | F(2, 85) = 1.00, | 0.27 [-2.08, 2.62] | 0.93 [-1.30, 3.16] | 1.20 [-1.00, 3.40] |
|  | AT | 30 | 90.60 | (4.07) | 88.81 | (4.21) | p = .372 |  |  |  |
|  | RT | 30 | 88.80 | (6.36) | 87.45 | (4.59) |  |  |  |  |
|  | WCG | 34 | 90.30 | (5.46) | 89.42 | (4.84) |  |  |  |  |
| Sleep Latency |  |  |  |  |  |  | F(2, 67) = 0.26, | -0.15 [-2.91, 2.61] | -0.56 [-3.09, 1.98] | -0.70 [-3.32, 1.91] |
|  | AT | 26 | 5.81 | (6.92) | 5.64 | (4.86) | p = .773 |  |  |  |
|  | RT | 23 | 4.33 | (3.25) | 4.99 | (4.12) |  |  |  |  |
|  | WCG | 27 | 5.07 | (8.30) | 4.73 | (2.83) |  |  |  |  |
| Wake after Sleep Onset |  |  |  |  |  |  | F(2, 85) = 1.71,  p = .187 | -2.02 [-14.93, 10.89] | -6.63 [-18.93, 5.68] | -8.64 [-20.73, 3.45] |
|  | AT | 30 | 45.23 | (19.43) | 55.30 | (22.20) |  |  |  |  |
|  | RT | 30 | 52.34 | (27.41) | 62.08 | (24.91) |  |  |  |  |
|  | WCG | 34 | 46.32 | (27.47) | 50.51 | (25.79) |  |  |  |  |
| Number of Awakenings |  |  |  |  |  |  | F(2, 85) = 0.78,  p = .464 | -1.19 [-3.86, 1.48] | 0.07 [-2.48, 2.63] | -1.12 [-3.64, 1.40] |
|  | AT | 30 | 13.42 | (4.57) | 15.53 | (4.97) |  |  |  |  |
|  | RT | 30 | 14.72 | (5.78) | 17.42 | (4.86) |  |  |  |  |
|  | WCG | 34 | 12.92 | (5.11) | 15.22 | (5.03) |  |  |  |  |
| Length of Awakenings |  |  |  |  |  |  | F(2, 85) = 1.02,  p = .365 | 0.08 [-0.62, 0.78] | -0.36 [-1.03, 0.30] | -0.28 [-0.94, 0.37] |
|  | AT | 30 | 3.56 | (1.92) | 3.65 | (1.51) |  |  |  |  |
|  | RT | 30 | 3.63 | (1.49) | 3.67 | (1.13) |  |  |  |  |
|  | WCG | 34 | 3.55 | (1.32) | 3.37 | (1.27) |  |  |  |  |

*Note:* All values represent the subset of participants that were integrated in the respective analysis.

AT: Aerobic Training Group; CI: Confidence Interval; RT: Resistance Training Group; SD: standard deviation; WCG: Waitlist Control Group

^a^ Unadjusted mean values

^b^ Group differences based on analyses of coviarance (ANCOVA) adjusted for baseline value of the outcome, tumor type (HR-, HER2+/HR-, and HR+/HER2-), and the treatment following the surgery until T3 (i.e., chemotherapy, radiotherapy, targeted therapies); Post-hoc comparisons between groups were adjusted using Bonferroni correction.
