# Supplementary material for: Effects of aerobic or resistance exercise on sleep and cancer-related fatigue in patients with breast cancer during or after neoadjuvant chemotherapy: a 3-arm randomized controlled trial
Source: BMC Med. 2026 Jan 28;24:114. doi: 10.1186/s12916-026-04669-3 (PMC12924517; doi:10.1186/s12916-026-04669-3)
Supplement: Supplementary file 5 — Additional file 5. Table S5: Group differences between resistance training after surgeryand aerobic trainingand resistance trainingbefore surgery in sleep and fatigue parameters 12 months post-surgery [file 12916_2026_4669_MOESM5_ESM.docx]

**Table S5.** Group differences between resistance training after surgery (WCG) and aerobic training (AT) and resistance training (RT) before surgery in sleep and fatigue parameters 12 months post-surgery (T4)

| Outcome | Range | Group | N | T0: Baseline | | T4: 12 months  post-surgery | | Overall group  effect | AT vs. RT | WCG vs. AT | WCG vs. RT |
| --- | --- | --- | --- | --- | --- | --- | --- | --- | --- | --- | --- |
|  |  |  |  | Mean | (SD) | Mean | (SD) | F(df1, df2), p | Mean difference  [95% CI]^b^ | Mean difference  [95% CI]^b^ | Mean difference [95% CI]^b^ |
| PSQI:  Global sleep score | 0-21 |  |  |  |  |  |  | F(2, 107) = 0.21, p = .812 | -0.01 [-1.84, 1.82] | 0.44 [-1.42, 2.29] | 0.43 [-1.45, 2.30] |
|  |  | AT | 41 | 5.46 | (3.03) | 6.07 | (3.56) |  |  |  |  |
|  |  | RT | 38 | 6.53 | (3.22) | 6.58 | (3.67) |  |  |  |  |
|  |  | WCG | 37 | 6.81 | (3.39) | 6.95 | (3.53) |  |  |  |  |
| PSQI subscales |  |  |  |  |  |  |  |  |  |  |  |
| Sleep Quality | 0-3 |  |  |  |  |  |  | F(2, 124) = 1.90, | 0.13 [-0.22, 0.48] | -0.28 [-0.62, 0.07] | -0.15 [-0.50, 0.20] |
|  |  | AT | 45 | 1.07 | (0.72) | 1.33 | (0.77) | p = .154 |  |  |  |
|  |  | RT | 41 | 1.24 | (0.62) | 1.32 | (0.76) |  |  |  |  |
|  |  | WCG | 47 | 1.43 | (0.64) | 1.23 | (0.72) |  |  |  |  |
| Sleep latency | 0-3 |  |  |  |  |  |  | F(2, 120) = 0.99, | 0.15 [-0.33, 0.63] | -0.27 [-0.74, 0.20] | -0.12 [-0.60, 0.36] |
|  |  | AT | 45 | 0.91 | (0.93) | 1.20 | (1.10) | p = .374 |  |  |  |
|  |  | RT | 40 | 1.20 | (1.02) | 1.23 | (0.97) |  |  |  |  |
|  |  | WCG | 44 | 1.32 | (1.07) | 1.09 | (1.03) |  |  |  |  |
| Sleep duration | 0-3 |  |  |  |  |  |  | F(2, 124) = 0.72, | 0.00 [-0.40, 0.39] | 0.17 [-0.22, 0.55] | 0.16 [-0.23, 0.56] |
|  |  | AT | 47 | 0.60 | (0.74) | 0.51 | (0.69) | p = .487 |  |  |  |
|  |  | RT | 41 | 0.71 | (0.78) | 0.56 | (0.67) |  |  |  |  |
|  |  | WCG | 45 | 0.78 | (0.82) | 0.76 | (0.93) |  |  |  |  |
| Sleep efficiency | 0-3 |  |  |  |  |  |  | F(2, 124) = 0.49, | 0.15 [-0.37, 0.67] | 0.06 [-0.45, 0.56] | 0.21 [-0.32, 0.73] |
|  |  | AT | 47 | 0.96 | (1.04) | 1.04 | (1.06) | p = .616 |  |  |  |
|  |  | RT | 41 | 1.10 | (1.14) | 0.95 | (0.95) |  |  |  |  |
|  |  | WCG | 45 | 1.00 | (0.98) | 1.09 | (1.16) |  |  |  |  |
| Sleep disturbances | 0-3 |  |  |  |  |  |  | F(2, 124) = 0.49, | -0.05 [-0.32, 0.22] | 0.23 [-0.03, 0.50] | 0.18 [-0.09, 0.46] |
|  |  | AT | 45 | 1.04 | (0.42) | 1.07 | (0.54) | p = .616 |  |  |  |
|  |  | RT | 40 | 1.15 | (0.30) | 1.15 | (0.48) |  |  |  |  |
|  |  | WCG | 44 | 1.25 | (0.62) | 1.34 | (0.57) |  |  |  |  |
| Daytime dysfunction | 0-3 |  |  |  |  |  |  | F(2, 124) = 1.91, p = .153 | -0.10 [-0.51, 0.31] | 0.31 [-0.08, 0.70] | 0.21 [-0.19, 0.61] |
|  |  | AT | 45 | 0.73 | (0.69) | 0.87 | (0.69) |  |  |  |  |
|  |  | RT | 41 | 0.73 | (0.59) | 0.90 | (0.83) |  |  |  |  |
|  |  | WCG | 47 | 0.79 | (0.59) | 1.17 | (0.84) |  |  |  |  |

**Table S5.** (continued)

| Outcome | Range | Group | N | T0: Baseline | | T4: 12 months  post-surgery | | Overall group  effect | AT vs. RT | WCG vs. AT | WCG vs. RT |
| --- | --- | --- | --- | --- | --- | --- | --- | --- | --- | --- | --- |
|  |  |  |  | Mean | (SD) | Mean | (SD) | F(df1, df2), p | Mean difference [95% CI]^b^ | Mean difference [95% CI]^b^ | Mean difference [95% CI]^b^ |
| Fatigue dimensions |  |  |  |  |  |  |  |  |  |  |  |
| Total  Fatigue | 0-100 |  |  |  |  |  |  | F(2, 126) = 0.04, p = .964 | -0.45 [-11.00, 10.09] | -0.69 [-10.96, 9.57] | -1.15 [-11.52, 9.22] |
|  |  | AT | 45 | 17.18 | (16.41) | 21.11 | (19.22) |  |  |  |  |
|  |  | RT | 42 | 20.69 | (15.82) | 23.08 | (24.92) |  |  |  |  |
|  |  | WCG | 48 | 24.83 | (19.31) | 25.32 | (23.10) |  |  |  |  |
| Physical  Fatigue | 0-100 |  |  |  |  |  |  | F(2, 126) = 0.09, p = .914 | -0.76 [-14.75, 13.22] | 2.32 [-11.28, 15.92] | 1.56 [-12.15, 15.27] |
|  |  | AT | 45 | 21.26 | (22.38) | 29.48 | (23.07) |  |  |  |  |
|  |  | RT | 42 | 27.30 | (21.17) | 32.06 | (30.00) |  |  |  |  |
|  |  | WCG | 48 | 30.97 | (25.45) | 36.53 | (30.41) |  |  |  |  |
| Emotional  Fatigue | 0-100 |  |  |  |  |  |  | F(2, 126) = 0.06, p = .941 | 1.16 [-11.11, 13.42] | -1.67 [-13.49, 10.15] | -0.51 [-12.54, 11.52] |
|  |  | AT | 45 | 23.21 | (23.55) | 18.77 | (25.71) |  |  |  |  |
|  |  | RT | 42 | 24.34 | (23.95) | 18.25 | (27.96) |  |  |  |  |
|  |  | WCG | 48 | 28.94 | (28.88) | 19.68 | (23.51) |  |  |  |  |
| Cognitive  Fatigue | 0-100 |  |  |  |  |  |  | F(2, 125) = 0.14, p = .866 | 1.48 [-8.48, 11.43] | -2.05 [-11.50, 7.41] | -0.57 [-10.26, 9.12] |
|  |  | AT | 45 | 7.41 | (17.61) | 10.74 | (18.85) |  |  |  |  |
|  |  | RT | 41 | 11.38 | (13.14) | 11.79 | (21.81) |  |  |  |  |
|  |  | WCG | 48 | 10.07 | (16.39) | 10.42 | (19.64) |  |  |  |  |

*Note:* Baseline values represent the subset of participants with available data at T4; No analyses were calculated for the Sleep Medication scale from the Pittsburgh Sleep Quality Inventory due to few cases (overall n=12 with value > 0); AT: Aerobic Training Group; CI: Confidence Interval; PSQI: Pittsburgh Sleep Quality Inventory; RT: Resistance Training Group; SD: standard deviation; WCG: Waitlist Control Group

^a^ Unadjusted mean values

^b^ Group differences based on analyses of coviarance (ANCOVA) adjusted for baseline value of the outcome and tumor type (HR-, HER2+/HR-, and HR+/HER2-), and the treatment following T2 (i.e., chemotherapy, radiotherapy, targeted therapies); Post-hoc comparisons between groups were adjusted using Bonferroni Correction.
