# Supplementary material for: Effects of aerobic or resistance exercise on sleep and cancer-related fatigue in patients with breast cancer during or after neoadjuvant chemotherapy: a 3-arm randomized controlled trial
Source: BMC Med. 2026 Jan 28;24:114. doi: 10.1186/s12916-026-04669-3 (PMC12924517; doi:10.1186/s12916-026-04669-3)
Supplement: Supplementary file 6 — Additional file 6. Table S6: Moderation analyses: interaction effects on global sleep score and total fatigue at T2 and T3 by symptom burden at baseline and NACT agents [file 12916_2026_4669_MOESM6_ESM.docx]

**Table S6.** Moderation analyses: interaction effects on global sleep score and total fatigue at T2 and T3 by symptom burden at baseline and NACT agents

|  | **Global Sleep Score (T2)** | | | **Total Fatigue (T2)** | | | **Global Sleep Score (T3)** | | | | **Total Fatigue (T3)** | | | |  |
| --- | --- | --- | --- | --- | --- | --- | --- | --- | --- | --- | --- | --- | --- | --- | --- |
| **Moderator** | **n** | **F** | **p** | **n** | **F** | **p** | | **n** | **F** | **p** | | **n** | **F** | **p** | |
| Symptom burden at baseline |  |  |  |  |  |  | |  |  |  | |  |  |  | |
| Emotional distress^a^ | 128 | 0.02 | .984 | 151 | 0.20 | .816 | | 121 | 0.38 | .685 | | 138 | 2.35 | .099 | |
| Global sleep quality^b^ | 135 | 0.16 | .853 | 141 | 0.07 | .929 | | 129 | 0.09 | .916 | | 134 | 0.75 | .473 | |
| Total fatigue^c^ | 133 | 0.07 | .930 | 161 | 1.11 | .331 | | 127 | 0.91 | .404 | | 148 | 1.46 | .236 | |
| NACT agents^d^ |  |  |  |  |  |  | |  |  |  | |  |  |  | |
| Platinum | 135 | 3.31 | .040 | 161 | 0.18 | .837 | | 129 | 0.92 | .400 | | 148 | 1.41 | .247 | |
| Cyclophosphamide | 135 | 0.14 | .871 | 161 | 0.01 | .992 | | 129 | 0.88 | .420 | | 148 | 0.92 | .402 | |
| Anthracyclines | 135 | 0.16 | .850 | 161 | 0.15 | .865 | | 129 | 1.32 | .271 | | 148 | 0.20 | .822 | |

*Note:* Shown are F statistics and *p*-values of group × moderator terms from the same ANCOVA models as in the main analyses predicting each outcome at T2 or T3, adjusting for the outcome at baseline and tumor type; T3 models additionally adjusted for chemotherapy, radiotherapy, and hormonal therapy received between T2 and T3. N’s denotes the model-wise complete-case sample size and therefore varies by moderator and time point.

^a^ Baseline emotional distress (PHQ-4): no = < 3; at least mild = ≥ 3

^b^ Baseline global sleep quality (PSQI global score): good = ≤ 5; poor = > 5

^c^ Baseline total fatigue (EORTC QLQ-FA12): low = < 33.3; high = ≥ 33.3

ᵈ NACT agents: 1 = NACT included this agent; 0 = NACT did not include this agent

*Abbreviations*: EORTC QLQ-FA12 = European Organisation for Research and Treatment of Cancer Quality of Life Questionnaire - Fatigue Module; NACT = Neoadjuvant chemotherapy. PHQ-4: Patient Health Questionnaire-4; PSQI = Pittsburgh Sleep Quality Index (global score).
