# Supplementary material for: Effects of aerobic or resistance exercise on sleep and cancer-related fatigue in patients with breast cancer during or after neoadjuvant chemotherapy: a 3-arm randomized controlled trial
Source: BMC Med. 2026 Jan 28;24:114. doi: 10.1186/s12916-026-04669-3 (PMC12924517; doi:10.1186/s12916-026-04669-3)
Supplement: Supplementary file 7 — Additional file 7. Table S7a-S7b. Table S7a: Group differences between aerobic trainingand resistance trainingbefore surgery and resistance training after surgeryin sleep and fatigue parameters at post-interventionwith missing data imputation. Table S7b: Group differences between resistance training after surgeryand aerobic trainingand resistance trainingbefore surgery in sleep and fatigue parameters at 6 months post-surgerywith missing data imputation [file 12916_2026_4669_MOESM7_ESM.docx]

**Table S7a.** Group differences between aerobic training (AT) and resistance training (RT) before surgery and resistance training after surgery (WCG) in sleep and fatigue parameters at post-intervention (T2) with missing data imputation

| Outcome | Range | Group | N | T0: Baseline | T2: Post-intervention | Overall group  effect | AT vs. RT | AT vs. WCG | RT vs. WCG |
| --- | --- | --- | --- | --- | --- | --- | --- | --- | --- |
|  |  |  |  | Mean^a^ (SD) | Mean^a^ (SD) | F(df1, df2), p | Mean difference [95% CI]^b^ | Mean difference [95% CI]^b^ | Mean difference  [95% CI]^b^ |
| PSQI:  Global score | 0-21 |  |  |  |  | F(2, 178) = 0.53, p = 0.591 | 0.09 [-1.21, 1.39] | 0.30 [-0.98, 1.58] | 0.21 [-1.11, 1.53] |
|  |  | AT | 62 | 5.56 (2.93) | 6.64 (3.34) |  |  |  |  |
|  |  | RT | 62 | 6.60 (3.29) | 7.04 (3.43) |  |  |  |  |
|  |  | WCG | 60 | 6.54 (3.46) | 6.77 (3.62) |  |  |  |  |
| PSQI subscales | |  |  |  |  |  |  |  |  |
| Sleep quality | 0-3 |  |  |  |  | F(2, 178) = 0.32 | 0.09 [-0.17, 0.35] | 0.20 [-0.07, 0.47] | 0.11 [-0.15, 0.37] |
|  |  | AT | 62 | 1.07 (0.68) | 1.35 (0.78) | p = .727 |  |  |  |
|  |  | RT | 62 | 1.27 (0.61) | 1.36 (0.67) |  |  |  |  |
|  |  | WCG | 60 | 1.40 (0.64) | 1.32 (0.71) |  |  |  |  |
| Sleep latency | 0-3 |  |  |  |  | F(2, 178) = 1.32, | 0.10 [-0.27, 0.48] | 0.16 [-0.22, 0.54] | 0.06 [-0.32, 0.44] |
|  |  | AT | 62 | 0.92 (0.89) | 1.26 (1.04) | p = .271 |  |  |  |
|  |  | RT | 62 | 1.26 (1.06) | 1.31 (1.00) |  |  |  |  |
|  |  | WCG | 60 | 1.26 (1.06) | 1.25 (1.00) |  |  |  |  |
| Sleep duration^c^ | 0-3 |  |  |  |  | F(2, 178) = 1.36 | -0.06 [-0.24, 0.11] | -0.14 [-0.31, 0.04] | -0.07 [-0.24, 0.10] |
|  |  | AT | 62 | 0.39 (0.43) | 0.23 (0.41) | p = .259 |  |  |  |
|  |  | RT | 62 | 0.48 (0.46) | 0.33 (0.48) |  |  |  |  |
|  |  | WCG | 60 | 0.43 (0.45) | 0.38 (0.49) |  |  |  |  |
| Sleep efficiency | 0-3 |  |  |  |  | F(2, 178) = 0.10 | -0.09 [-0.53, 0.36] | 0.01 [-0.43, 0.44] | 0.10 [-0.34, 0.54] |
|  |  | AT | 62 | 0.95 (1.04) | 1.06 (1.16) | p = .909 |  |  |  |
|  |  | RT | 62 | 1.14 (1.12) | 1.23 (1.13) |  |  |  |  |
|  |  | WCG | 60 | 0.98 (1.01) | 1.05 (1.24) |  |  |  |  |
| Sleep disturbances | 0-3 |  |  |  |  | F(2, 178) =0.06, | 0.06 [-0.14, 0.26] | 0.03 [-0.17, 0.22] | -0.03 [-0.23, 0.17] |
|  |  | AT | 62 | 1.11 (0.53) | 1.30 (0.50) | p = .938 |  |  |  |
|  |  | RT | 62 | 1.12 (0.47) | 1.25 (0.52) |  |  |  |  |
|  |  | WCG | 60 | 1.21 (0.58) | 1.29 (0.53) |  |  |  |  |
| Daytime dysfunction | 0-3 |  |  |  |  | F(2, 178) =0.14,  p = .872 | 0.10 [-0.16, 0.36] | 0.12 [-0.14, 0.38] | 0.01 [-0.24, 0.27] |
|  |  | AT | 62 | 0.74 (0.67) | 1.22 (0.67) |  |  |  |  |
|  |  | RT | 62 | 0.73 (0.61) | 1.11 (0.60) |  |  |  |  |
|  |  | WCG | 60 | 0.83 (0.59) | 1.14 (0.72) |  |  |  |  |

**Table S7a.** (continued)

| Outcome | Range | Group | T0: Baseline | | T2: Post-intervention | Overall group  effect | AT vs. RT | AT vs. WCG | RT vs. WCG |
| --- | --- | --- | --- | --- | --- | --- | --- | --- | --- |
|  |  |  | N | Mean^a^ (SD) | Mean^a^ (SD) | F(df1, df2), p | Mean difference [95% CI]^b^ | Mean difference [95% CI]^b^ | Mean difference [95% CI]^b^ |
| Fatigue dimensions | |  |  |  |  |  |  |  |  |
| Total Fatigue | 0-100 |  |  |  |  | F(2, 178) = 1.95,  p = .145 | -1.20 [-8.82, 6.42] | 3.78 [-3.94, 11.50] | 4.98 [-2.85, 12.81] |
|  |  | AT | 62 | 19.14 (17.65) | 30.39 (19.57) |  |  |  |  |
|  |  | RT | 62 | 20.71 (15.90) | 32.29 (21.47) |  |  |  |  |
|  |  | WCG | 60 | 25.65 (19.23) | 30.08 (21.67) |  |  |  |  |
| Physical Fatigue | 0-100 |  |  |  |  | F(2, 178) = 0.41,  p = .663 | 0.67 [-9.61, 10.95] | 4.52 [-6.10, 15.14] | 3.85 [-6.21, 13.91] |
|  |  | AT | 62 | 23.54 (23.21) | 45.21 (26.31) |  |  |  |  |
|  |  | RT | 62 | 26.30 (20.05) | 45.37 (25.85) |  |  |  |  |
|  |  | WCG | 60 | 32.19 (24.51) | 43.80 (27.68) |  |  |  |  |
| Emotional Fatigue | 0-100 |  |  |  |  | F(2, 178) = 0.99,  p = .375 | -3.91 [-13.26, 5.45] | 1.60 [-7.99, 11.19] | 5.50 [-4.14, 15.14] |
|  |  | AT | 62 | 24.98 (24.23) | 22.22 (23.88) |  |  |  |  |
|  |  | RT | 62 | 24.55 (23.67) | 25.98 (28.25) |  |  |  |  |
|  |  | WCG | 60 | 29.08 (28.87) | 22.55 (24.83) |  |  |  |  |
| Cognitive Fatigue | 0-100 |  |  |  |  | F(2, 178) = 0.09,  p = .911 | -0.26 [-8.73, 8.22] | 0.07 [-8.57, 8.71] | 0.32 [-7.99, 8.64] |
|  |  | AT | 62 | 10.83 (19.59) | 14.94 (22.42) |  |  |  |  |
|  |  | RT | 62 | 12.14 (15.04) | 15.88 (23.38) |  |  |  |  |
|  |  | WCG | 60 | 11.01 (17.94) | 15.18 (23.89) |  |  |  |  |

*Note:* No analyses were calculated for the Sleep Medication scale from the Pittsburgh Sleep Quality Inventory due to few cases (overall n=14 with value > 0); AT: Aerobic Training Group; CI: Confidence Interval; PSQI: Pittsburgh Sleep Quality Inventory; RT: Resistance Training Group; SD: standard deviation; WCG: Waitlist Control Group

^a^ Group means are pooled across 30 multiply imputed datasets generated with Multiple Imputation by Chained Equations

^b^ Group differences come from ANCOVA models fitted in each imputed dataset and pooled with Rubin’s rules, adjusted for the baseline value of the outcome and tumor type.

^c^ Variable was transformed to its natural logarithm

**Table S7b.** Group differences between resistance training after surgery (WCG) and aerobic training (AT) and resistance training (RT) before surgery in sleep and fatigue parameters at 6 months post-surgery (T3) with missing data imputation

| Outcome | Range | Group | N | T0: Baseline | T3: 6-month post-surgery | Overall group  effect | AT vs. RT | WCG vs. AT | WCG vs. RT |
| --- | --- | --- | --- | --- | --- | --- | --- | --- | --- |
|  |  |  |  | Mean^a^ (SD) | Mean^a^ (SD) | F(df1, df2), p | Mean difference  [95% CI]^b^ | Mean difference [95% CI]^b^ | Mean difference  [95% CI]^b^ |
| PSQI:  Global score^c^ | 0-21 |  |  |  |  | F(2, 175) = 2.73, p = 0.068 | 0.10 [-0.08, 0.28] | **-0.21 [-0.40, -0.02]** | -0.11 [-0.30, 0.08] |
|  |  | AT | 62 | 1.76 (0.53) | 1.98 (0.49) |  |  |  |  |
|  |  | RT | 62 | 1.91 (0.52) | 1.96 (0.52) |  |  |  |  |
|  |  | WCG | 60 | 1.89 (0.55) | 1.83 (0.56) |  |  |  |  |
| PSQI subscales |  |  |  |  |  |  |  |  |  |
| Sleep quality | 0-3 |  |  |  |  | F(2, 175) = 2.68, | -0.15 [-0.42, 0.11] | -0.16 [-0.44, 0.11] | **-0.32 [-0.58, -0.06]** |
|  |  | AT | 62 | 1.07 (0.68) | 1.24 (0.73) | p = 0.072 |  |  |  |
|  |  | RT | 62 | 1.26 (0.62) | 1.52 (0.78) |  |  |  |  |
|  |  | WCG | 60 | 1.39 (0.64) | 1.24 (0.66) |  |  |  |  |
| Sleep latency | 0-3 |  |  |  |  | F(2, 175) = 4.56, | 0.44 [0.05, 0.83] | **-0.55 [-0.94, -0.16]** | -0.11 [-0.52, 0.31] |
|  |  | AT | 62 | 0.91 (0.88) | 1.50 (1.05) | p = 0.012 |  |  |  |
|  |  | RT | 62 | 1.24 (1.05) | 1.23 (1.08) |  |  |  |  |
|  |  | WCG | 60 | 1.26 (1.06) | 1.13 (1.02) |  |  |  |  |
| Sleep duration^c^ | 0-3 |  |  |  |  | F(2, 175) = 0.12, | 0.04 [-0.14, 0.22] | 0.03 [-0.15, 0.21] | 0.07 [-0.11, 0.25] |
|  |  | AT | 62 | 0.39 (0.43) | 0.33 (0.47) | p = 0.890 |  |  |  |
|  |  | RT | 62 | 0.47 (0.46) | 0.34 (0.44) |  |  |  |  |
|  |  | WCG | 60 | 0.43 (0.45) | 0.38 (0.48) |  |  |  |  |
| Sleep efficiency | 0-3 |  |  |  |  | F(2, 175) = 1.39, | 0.13 [-0.31, 0.57] | -0.24 [-0.67, 0.19] | -0.11 [-0.52, 0.30] |
|  |  | AT | 62 | 0.95 (1.04) | 1.18 (1.11) | p = 0.253 |  |  |  |
|  |  | RT | 62 | 1.12 (1.12) | 1.17 (1.14) |  |  |  |  |
|  |  | WCG | 60 | 0.97 (1.01) | 0.96 (1.14) |  |  |  |  |
| Sleep disturbances | 0-3 |  |  |  |  | F(2, 175) = 0.71, | 0.09 [-0.13, 0.32] | -0.13 [-0.35, 0.08] | -0.04 [-0.27, 0.18] |
|  |  | AT | 62 | 1.11 (0.53) | 1.32 (0.52) | p = 0.494 |  |  |  |
|  |  | RT | 62 | 1.12 (0.47) | 1.22 (0.52) |  |  |  |  |
|  |  | WCG | 60 | 1.21 (0.59) | 1.19 (0.57) |  |  |  |  |
| Daytime dysfunction | 0-3 |  |  |  |  | F(2, 175) = 2.34, | 0.03 [-0.28, 0.35] | -0.24 [-0.55, 0.07] | -0.21 [-0.50, 0.09] |
|  |  | AT | 62 | 0.75 (0.67) | 1.01 (0.78) | p = 0.100 |  |  |  |
|  |  | RT | 62 | 0.73 (0.61) | 0.98 (0.73) |  |  |  |  |
|  |  | WCG | 60 | 0.83 (0.60) | 0.80 (0.85) |  |  |  |  |

**Table S7b.** (continued)

| Outcome | Range | Group | N | T0: Baseline | T3: 6-month post-surgery | Overall group  effect | AT vs. RT | WCG vs. AT | WCG vs. RT |
| --- | --- | --- | --- | --- | --- | --- | --- | --- | --- |
|  |  |  |  | Mean^a^ (SD) | Mean^a^ (SD) | F(df1, df2), p | Mean difference  [95% CI]^b^ | Mean difference  [95% CI]^b^ | Mean difference  [95% CI]^b^ |
| Fatigue dimensions | |  |  |  |  |  |  |  |  |
| Total  Fatigue | 0-100 |  |  |  |  | F(2, 175) = 5.32, p = 0.006 | 1.55 [-6.03, 9.13] | **-9.40 [-16.99, -1.81]** | -7.85 [-15.66, -0.04] |
|  |  | AT | 62 | 19.16 (17.68) | 24.95 (19.75) |  |  |  |  |
|  |  | RT | 62 | 20.74 (15.87) | 24.51 (22.54) |  |  |  |  |
|  |  | WCG | 60 | 25.56 (19.29) | 19.31 (20.49) |  |  |  |  |
| Physical Fatigue | 0-100 |  |  |  |  | F(2, 175) = 2.89, p = 0.058 | 2.54 [-8.72, 13.80] | **-12.21 [-22.19, -2.23**] | -9.67 [-20.60, 1.25] |
|  |  | AT | 62 | 23.61 (23.37) | 35.41 (24.82) |  |  |  |  |
|  |  | RT | 62 | 26.38 (19.91) | 34.42 (26.91) |  |  |  |  |
|  |  | WCG | 60 | 32.44 (24.66) | 27.11 (25.80) |  |  |  |  |
| Emotional Fatigue | 0-100 |  |  |  |  | F(2, 175) = 1.04, p = 0.354 | -0.45 [-9.30, 8.39] | -4.73 [-13.53, 4.08] | -5.18 [-14.26, 3.90] |
|  |  | AT | 62 | 24.91 (24.12) | 19.56 (23.17) |  |  |  |  |
|  |  | RT | 62 | 24.62 (23.72) | 20.55 (25.71) |  |  |  |  |
|  |  | WCG | 60 | 29.06 (28.91) | 16.96 (23.52) |  |  |  |  |
| Cognitive Fatigue | 0-100 |  |  |  |  | F(2, 175) = 0.59, p = 0.554 | 1.34 [-6.24, 8.92] | -2.99 [-10.36, 4.39] | -1.64 [-9.21, 5.92] |
|  |  | AT | 62 | 10.81 (19.76) | 12.09 (18.62) |  |  |  |  |
|  |  | RT | 62 | 12.16 (15.06) | 11.33 (21.04) |  |  |  |  |
|  |  | WCG | 60 | 10.98 (17.87) | 9.55 (18.31) |  |  |  |  |

*Note:* No analyses were calculated for the Sleep Medication scale from the Pittsburgh Sleep Quality Inventory due to few cases (overall *n*=17 with value > 0); 95% confidence intervals that do not include 0 are marked in bold; AT: Aerobic Training Group; CI: Confidence Interval; PSQI: Pittsburgh Sleep Quality Inventory; RT: Resistance Training Group; SD: Standard Deviation; WCG: Waitlist Control Group

^a^ Group means are pooled across 30 multiply imputed datasets generated with Multiple Imputation by Chained Equations

^b^ Group differences come from ANCOVA models fitted in each imputed dataset and pooled with Rubin’s rules, adjusted for the baseline value of the outcome, tumor type, and treatments received between T2 and T3 (chemotherapy, radiotherapy, hormonal therapy).

^c^ Variable was transformed to its natural logarithm
